# MANF serves as a novel hepatocyte factor to promote liver regeneration after 2/3 partial hepatectomy via doubly targeting Wnt/β-catenin signaling

**DOI:** 10.1038/s41419-024-07069-8

**Published:** 2024-09-18

**Authors:** Yanyan Liang, Qiong Mei, Enguang He, Petek Ballar, Chuansheng Wei, Yue Wang, Yue Dong, Jie Zhou, Xiaofang Tao, Wenyan Qu, Mingxia Zhao, Goma Chhetri, Limeng Wei, Juntang Shao, Yujun Shen, Jun Liu, Lijie Feng, Yuxian Shen

**Affiliations:** 1 School of Basic Medical Sciences, Anhui Medical University, Hefei, 230032 China; 2 Biopharmaceutical Research Institute, Anhui Medical University, Hefei, 230032 China; 3 Department of Biochemistry, Faculty of Pharmacy, Ege University, Izmir, 35100 Turkey; 4 Department of General Surgery, The First Affiliated Hospital, Anhui Medical University, Hefei, 230022 China

**Keywords:** Cell biology, Molecular biology

## Abstract

Liver regeneration is an intricate pathophysiological process that has been a subject of great interest to the scientific community for many years. The capacity of liver regeneration is very critical for patients with liver diseases. Therefore, exploring the mechanisms of liver regeneration and finding good ways to improve it are very meaningful. Mesencephalic astrocyte-derived neurotrophic factor (MANF), a member of newly identified neurotrophic factors (NTFs) family, extensively expresses in the liver and has demonstrated cytoprotective effects during ER stress and inflammation. However, the role of MANF in liver regeneration remains unclear. Here, we used hepatocyte-specific MANF knockout (MANF^Hep^^−/−^) mice to investigate the role of MANF in liver regeneration after 2/3 partial hepatectomy (PH). Our results showed that MANF expression was up-regulated in a time-dependent manner, and the peak level of mRNA and protein appeared at 24 h and 36 h after 2/3 PH, respectively. Notably, MANF knockout delayed hepatocyte proliferation, and the peak proliferation period was delayed by 24 h. Mechanistically, our in vitro results showed that MANF physically interacts with LRP5 and β-catenin, two essential components of Wnt/β-catenin pathway. Specifically, as a cofactor, MANF binds to the extracellular segment of LRP5 to activate Wnt/β-catenin signaling. On the other hand, MANF interacts with β-catenin to stabilize cytosolic β-catenin level and promote its nuclear translocation, which further enhance the Wnt/β-catenin signaling. We also found that MANF knockout does not affect the c-Met/β-catenin complex after 2/3 PH. In summary, our study confirms that MANF may serve as a novel hepatocyte factor that is closely linked to the activation of the Wnt/β-catenin pathway via intracellular and extracellular targets.

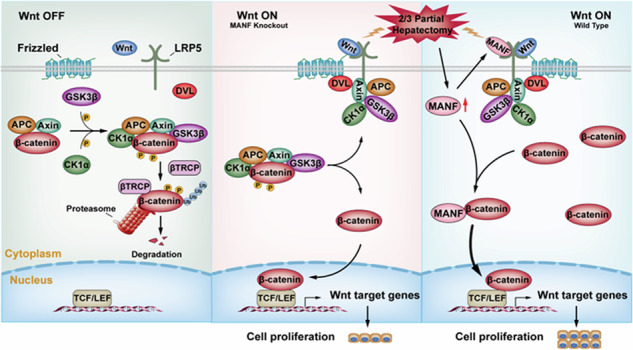

## Introduction

The liver’s unique ability to repair and regenerate has been widely recognized [1–3]. Mature hepatocytes are typically quiescent and highly differentiated, rarely dividing under normal conditions. DNA labeling studies have shown that the percentage of hepatocytes in DNA synthesis in the normal liver is very low, less than 0.2%. However, liver regeneration following acute injury is highly beneficial and has been extensively studied. The liver consists of various types of cells, hepatocytes accounting for approximately 80% of liver weight and about 70% of all liver cells [4]. As the primary functional cells of the liver, hepatocytes can proliferate under specific conditions, including surgical removal, chemical injury, and infection [1, 5, 6]. These cells can rapidly enter the cell cycle to restore liver mass and function.

As the liver’s ability to regenerate is crucial for repair and prognosis after partial hepatectomy and liver transplantation, there is an urgent need to gain a better understanding of the molecular and cellular mechanisms of liver regeneration following hepatectomy. 2/3 partial hepatectomy (PH) is a widely used and extensively studied model for liver regeneration in rodents [7]. After 2/3 PH, residual hepatocytes can quickly enter the cell cycle and undergo division to compensate for the lost liver tissues, restoring the original quality and size within a week in rodents [8, 9]. Clinically, liver regeneration carries important implications due to the treatment strategies for many liver diseases, such as liver fibrosis, liver tumor resection, and liver transplantation, all of which depend on the physiological and functional regeneration of the liver. Therefore, elucidating the potential mechanisms and physiological characteristics of liver regeneration may bring significant clinical benefits.

Neurotrophic factors (NTFs) are a class of molecules that promote the growth, survival, and differentiation of neurons, and also play a crucial role in regulating inflammation and facilitating tissue repair. Mesencephalic astrocyte-derived neurotrophic factor (MANF), previously termed ARMET, was identified in 2003 as a member of a new NTFs family that protects dopaminergic neurons [10]. MANF is located in the endoplasmic reticulum (ER) and is a secretory protein [10–12]. A growing body of research suggests that MANF has cellular protective effects and potential therapeutic application for a variety of diseases, including neurodegenerative diseases [13–16], cardiovascular diseases [17, 18], diabetes [19–21], retinal degeneration [22, 23], and liver injury [24–27].

In our previous studies, we found that MANF was extensively expressed in the liver, primarily in hepatocytes. Here, we used hepatocyte-specific MANF knockout (MANF^Hep−/−^) mice to investigate the role of MANF in liver regeneration. Our results showed that MANF expression was up-regulated in a time-dependent manner following 2/3 PH. Furthermore, we observed that MANF knockout displayed a delayed liver regeneration with cell cycle arrest. Based on our findings, we propose that MANF may represent a promising target for liver regeneration therapy, as it appears to function as a novel hepatocyte factor.

## Results

### MANF expression is up-regulated in a time-dependent manner after 2/3 PH

**Fig. 1:**
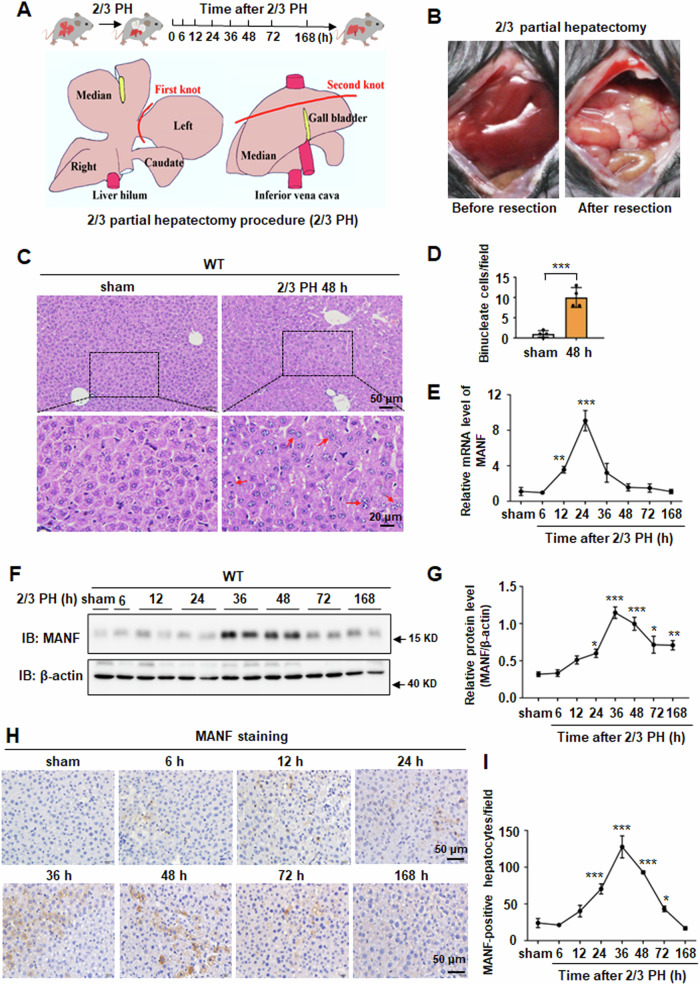
MANF expression is up-regulated in a time-dependent manner after 2/3 PH. **A**, **B** Schematic description of the experimental design for mice 2/3 partial hepatectomy (PH). Liver tissues were collected on sham, 6, 12, 24, 36, 48, 72, and 168 h after 2/3 PH. **C** Hematoxylin and eosin staining of livers slices of WT mice. The tissues were collected at 48 h after 2/3 PH. The sham group was used as a control. The binucleate cells were marked by red arrowheads. Scale bar = 50 μm (upper panel), scale bar = 20 μm (lower panel). **D** The quantitative data in panel C. Data were expressed as mean ± SEM, *n* = 4, ***P < 0.001. **E** MANF mRNA level was detected by qPCR assay. Data were expressed as mean ± SEM, *n* = 5, **P < 0.01, ***P < 0.001, compared with sham. **F** MANF protein level was detected by immunoblotting in WT mice livers after 2/3 PH. **G** The quantitative data in (**F**). Data were expressed as mean ± SEM, *n* = 7, *P < 0.05, **P < 0.01, ***P < 0.001, compared with sham. **H** MANF level was detected by immunohistochemical in WT mice livers after 2/3 PH. Scale bar = 50 μm. **I** The quantitative data in panel H. Data were expressed as mean ± SEM, *n* = 5, *P < 0.05, ***P < 0.001, compared with sham.

2/3 PH is a well-established model for studying liver regeneration. To determine whether MANF was involved in liver regeneration, wild-type (WT) and MANF^Hep−/−^ mice were subjected to 2/3 PH, and their liver tissues were analyzed at different time points after 2/3 PH (Fig. 1A, B). The results of hematoxylin and eosin staining showed that there was a significant increase in the number of binucleate cells in WT mice livers at 48 h after 2/3 PH, compared with sham group (Fig. 1C, D), indicating successful establishment of the model. MANF expression was then detected at different time points after 2/3 PH in WT mice. The result of qPCR analysis showed that the peak level of MANF mRNA was at 24 h after 2/3 PH (Fig. 1E). Immunoblotting analysis showed that MANF was up-regulated in a time-dependent manner, with the peak level at 36 h after 2/3 PH (Fig. 1F, G). The results from immunohistochemistry were consistent with those from immunoblotting (Fig. 1H, I). These findings suggest that 2/3 PH up-regulates MANF expression.

### Hepatocyte-specific MANF knockout delays the recovery of liver mass after 2/3 PH

**Fig. 2:**
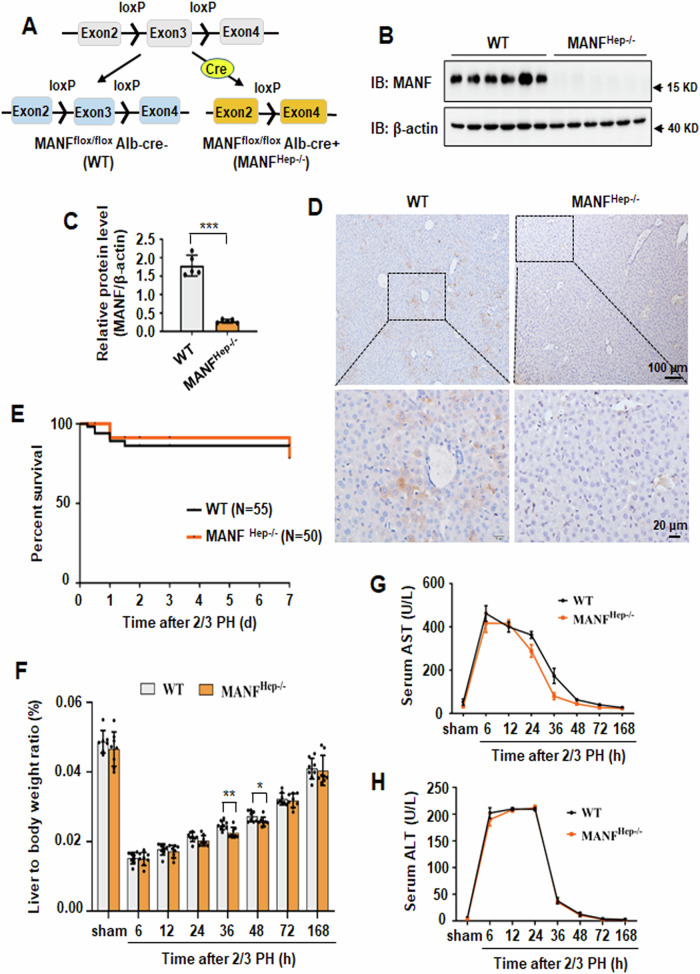
Hepatocyte-specific MANF knockout delays the recovery of liver mass after 2/3 PH. **A** Schematic description for hepatocyte-specific MANF knockout. MANF^flox/flox^ mice bear loxP sites flanking exon 3 of the *manf* gene on the C57BL/6 background. MANF^flox/flox^ mice were cross-bred with Alb-Cre mice to specifically knockout *manf* gene in hepatocytes (MANF^Hep−/−^ mice). **B** Efficiency of MANF knockout was confirmed by immunoblotting. **C** The quantitative data in panel B. Data were expressed as mean ± SEM, *n* = 6, ***P < 0.001. **D** Efficiency of MANF knockout was confirmed by immunohistochemistry. Scale bar = 100 μm (upper panel), scale bar = 20 μm (lower panel). **E** Kaplan–Meier estimates for the survival of WT and MANF^Hep−/−^ mice after 2/3 PH. **F** The ratio of liver weight to body weight in WT and MANF^Hep−/−^ mice after 2/3 PH. Data were expressed as mean ± SEM, *n* = 7–8, *P < 0.05, **P < 0.01, WT vs MANF^Hep−/−^. The time course of serum AST (**G**) and ALT (**H**) levels in mice after 2/3 PH. Data were expressed as mean ± SEM, *n* = 5, WT vs MANF^Hep−/−^.

We constructed hepatocyte-specific MANF knockout mice using Cre-loxP system. The mice that were homozygous for the loxP-flanked allele and positive for the Alb-cre transgene were referred to as MANF^Hep−/−^ (MANF^flox/flox^ Alb-cre + ) mice (Fig. 2A). The knockout efficiency of MANF in the hepatocytes was assessed by detecting the level of MANF in the liver tissues of WT and MANF^Hep−/−^ mice (Fig. 2B–D). No morphological differences were observed between WT and MANF^Hep−/−^ mice in the sham, 6, 12, and 24 h after 2/3 PH, as detected by hematoxylin and eosin staining (Supplementary Fig. S1). The postoperative survival rate was no significantly different between the two groups, and 2/3 PH was well-tolerated in both WT and MANF^Hep−/−^ mice (*P* = 0.394) (Fig. 2E). To assess whether MANF knockout had an effect on hepatocyte proliferation following 2/3 PH, the liver mass recovery was measured to evaluate the capacity of the liver regeneration. The liver weight to body weight ratio was increased gradually in both WT and MANF^Hep−/−^ mice after 2/3 PH over the time course of 0–7 days (Fig. 2F). Strikingly, compared with WT mice, the liver weight to body weight ratio was significantly reduced at 36 h and 48 h after 2/3 PH in MANF^Hep−/−^ mice (Fig. 2F). The levels of ALT and AST in the two groups were no significantly different after 2/3 PH (Fig. 2G, H). These findings suggest that MANF knockout delays the recovery of liver mass after 2/3 PH.

### Hepatocyte-specific MANF knockout leads to a delay in hepatocytes proliferation

**Fig. 3:**
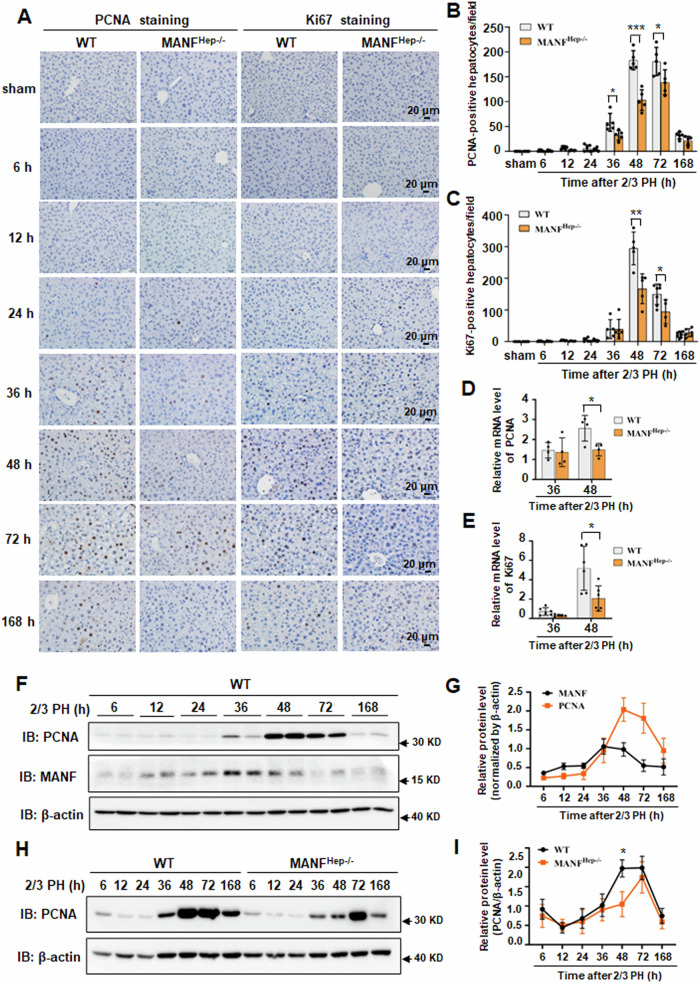
Hepatocyte-specific MANF knockout leads to a delay in hepatocytes proliferation after 2/3 PH. **A** PCNA and Ki67 in liver tissues at different time points after 2/3 PH were analyzed by immunohistochemistry. Scale bar = 20 μm. **B**, **C** The quantitative data in (**A**). Data were expressed as mean ± SEM, *n* = 5–6, *P < 0.05, **P < 0.01, ***P < 0.001, WT vs MANF^Hep−/−^. PCNA (**D**) and Ki67 (**E**) mRNA levels were detected by qPCR assay. Data were expressed as mean ± SEM, *n* = 5, *P < 0.05, WT vs MANF^Hep−/−^. **F** MANF and PCNA protein levels were detected by immunoblotting after 2/3 PH. **G** The time-course curves of MANF and PCNA protein levels quantitated according to the data in (**F**). Data were expressed as mean ± SEM, *n* = 3. **H** PCNA protein levels were detected by immunoblotting in WT and MANF^Hep−/−^ mice after 2/3 PH. **I** The quantitative data in panel H. Data were expressed as mean ± SEM, *n* = 3, *P < 0.05, WT *vs* MANF^Hep−/−^.

Mature hepatocytes are highly differentiated cells that rarely undergo cell division in normal condition. However, upon 2/3 PH, mature hepatocytes undergo proliferation to compensate for the lost liver mass and restore liver function [2, 9, 28]. We then investigated whether MANF is involved in hepatocytes proliferation after 2/3 PH by analyzing the expression of proliferation markers. The results of immunohistochemistry showed that PCNA and Ki67 expression were significantly increased at 36 h, and both peaked at 48 h, respectively in WT mice (Fig. 3A–C). However, PCNA expression was reached highest level at 72 h in MANF^Hep−/−^ mice (Fig. 3A, B). MANF^Hep−/−^ mice showed a significant decrease in the number of PCNA and Ki67-positive hepatocytes after 2/3 PH, compared with WT mice (Fig. 3A–C). The result of qPCR analysis showed that PCNA and Ki67 mRNA levels were attenuated at 48 h in MANF^Hep−/−^ mice (Fig. 3D, E). The results from immunoblotting were consistent with those from immunohistochemistry, which indicated that the proliferation peak of hepatocytes was delayed by 24 h in MANF^Hep−/−^ mice (Fig. 3F–I).

To confirm the effect of MANF on liver regeneration in vivo, we administrated His-tagged recombinant human MANF (rhMANF) to wild type (WT) mice by tail vein. The schedule of rhMANF administration was shown in Supplementary Fig. S2A. The presence of His-tagged rhMANF in the liver tissues was detected by using immunohistochemistry with anti-His antibody (Supplementary Fig. S2B), and the level of MANF in the liver tissues at the indicated time points after 2/3 PH was detected by using immunoblotting with anti-MANF antibody after rhMANF administration (Supplementary Fig. S2C–F). We found that MANF injection significantly promoted hepatocytes proliferation in WT mice after 2/3 PH (Supplementary Fig. S3A–C). We performed an additional experiment, in which the primary hepatocytes isolated from hepatocyte-specific MANF knockout (MANF^Hep−/−^) mice were treated with HGF, and then rhMANF was added to the primary hepatocytes for 12 h. We found that His-tagged MANF entered hepatocytes (Supplementary Fig. S3D) and increased PCNA level (Supplementary Fig. S3E). These findings suggest that hepatocyte MANF knockout delays hepatocyte proliferation.

### Hepatocyte-specific MANF knockout attenuates the cell cycle progression of hepatocytes

**Fig. 4:**
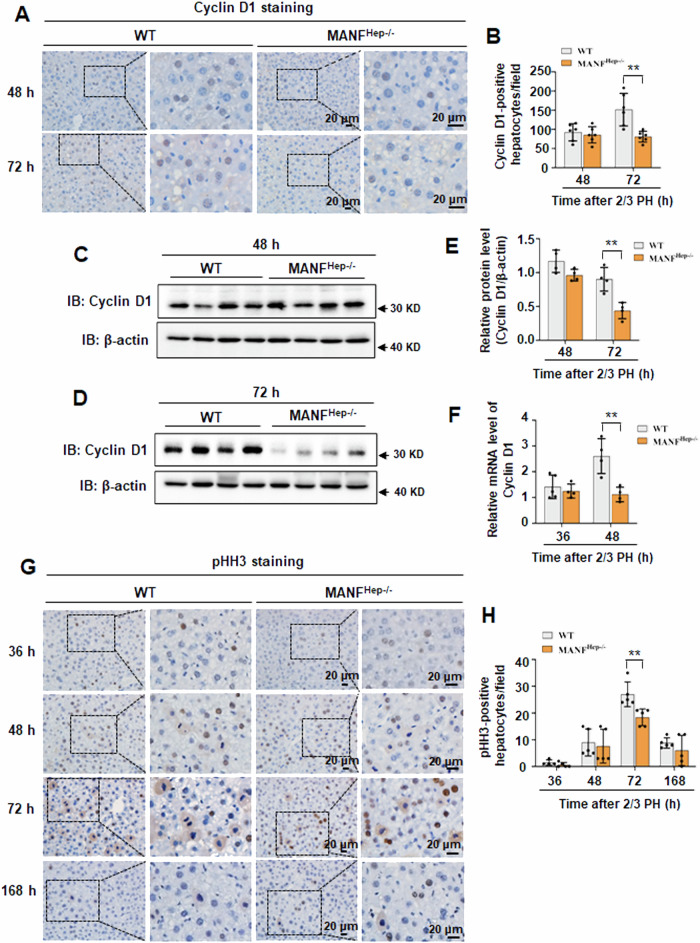
Hepatocyte-specific MANF knockout attenuates the cell cycle progression of hepatocytes. **A** Cyclin D1 in liver tissues at 48 h and 72 h after 2/3 PH were analyzed by immunohistochemistry. Scale bar = 20 μm. **B** The quantitative data in (**A**). Data were expressed as mean ± SEM, *n* = 5–6, *P < 0.05, **P < 0.01, WT vs MANF^Hep−/−^. Cyclin D1 protein level was detected by immunoblotting at 48 h (**C**) and 72 h (**D**) after 2/3 PH. **E** The quantitative data in (**C**, **D**). Data were expressed as mean ± SEM, *n* = 4, **P < 0.01, WT *vs* MANF^Hep−/−^. **F** Cyclin D1 mRNA level was detected by qPCR assay. Data were expressed as mean ± SEM, *n* = 4–5, **P < 0.01. **G** pHH3 level in liver tissues at different time points after 2/3 PH were analyzed by immunohistochemistry. Scale bar = 20 μm. **H** The quantitative data in (**G**). Data were expressed as mean ± SEM, *n* = 5, **P < 0.01, WT *vs* MANF^Hep−/−^.

To investigate whether MANF knockout affects DNA replication, we detected the expression of cell cycle-related proteins during regeneration. One of the earliest cell cycle events after 2/3 PH is the up-regulation of Cyclin D1 by extracellular mitogenic signals [8]. Immunohistochemistry of residual liver tissue after 2/3 PH revealed that Cyclin D1 expression was induced in both WT and MANF^Hep−/−^ mice, mainly in the nucleus of hepatocytes (Fig. 4A, B). Compared with WT mice, the number of Cyclin D1-positive hepatocytes were significantly reduced at 72 h in MANF^Hep−/−^ mice (Fig. 4A, B). To further quantify this difference, we evaluated the expression of Cyclin D1 protein level from immunoblotting and found significantly decreased at 72 h in MANF^Hep−/−^ mice (Fig. 4–E). The qPCR analysis showed that Cyclin D1 mRNA level was attenuated at 48 h in MANF^Hep−/−^ mice (Fig. 4F). As mitosis is an important index for evaluating proliferation, we also performed pHH3 staining to observe the mitosis of hepatocytes. The results showed that the mitosis of hepatocytes was significantly decreased at 72 h in MANF^Hep−/−^ mice (Fig. 4G, H). Taken together, these findings indicate that MANF knockout weakens the induction of Cyclin D1 and attenuates the cell cycle of hepatocytes.

### Hepatocyte-specific MANF knockout decreases β-catenin expression after 2/3 PH

**Fig. 5:**
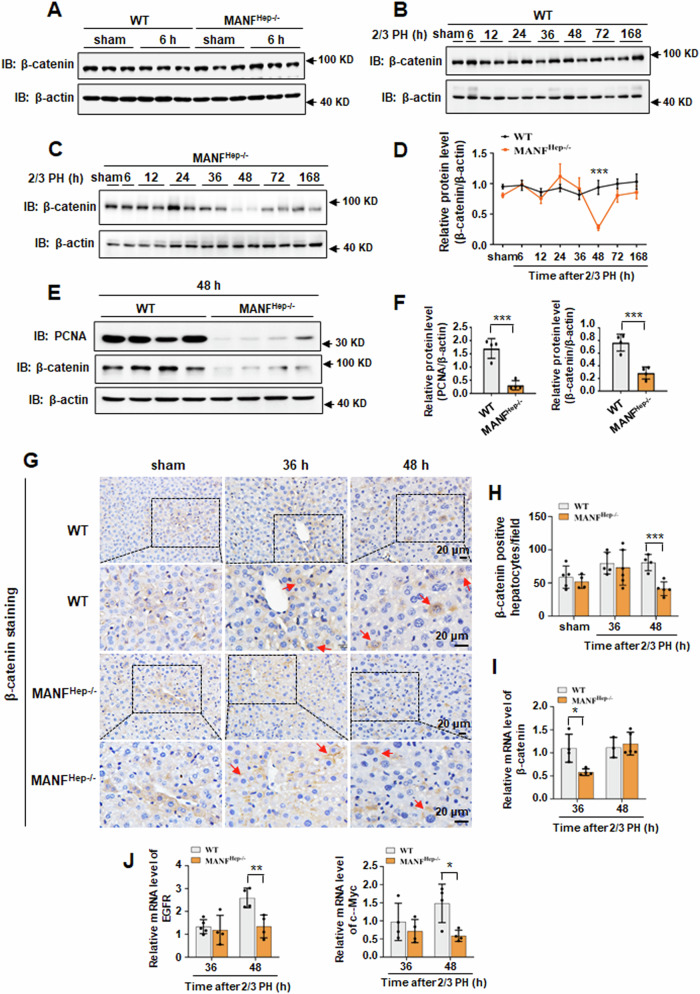
Hepatocyte-specific MANF knockout decreases β-catenin expression after 2/3 PH. **A** β-catenin protein level was detected by immunoblotting in the liver tissues of sham group and the operated mice at 6 h after 2/3 PH. β-catenin protein level was detected by immunoblotting in the liver tissues from WT (**B**) and MANF^Hep−/−^ (**C**) mice after 2/3 PH. **D** The quantitative data in (**A**–**C**). Data were expressed as mean ± SEM, *n* = 3, ***P < 0.001, WT *vs* MANF^Hep−/−^. **E** PCNA and β-catenin protein levels were detected by immunoblotting at 48 h after 2/3 PH. **F** The quantitative data in panel E. Data were expressed as mean ± SEM, n = 4, ***P < 0.001, WT vs MANF^Hep−/−^. **G** β-catenin level was detected by immunohistochemical in WT and MANF^Hep−/−^ mice after 2/3 PH. Scale bar = 20 μm. **H** The quantitative data in (**G**). Data were expressed as mean ± SEM, *n* = 5, ***P < 0.001, WT vs MANF^Hep−/−^. **I** β-catenin mRNA level was detected by qPCR assay at 36 h and 48 h after 2/3 PH. Data were expressed as mean ± SEM, *n* = 4–5, *P < 0.05, WT vs MANF^Hep−/−^. **J** c-Myc and EGFR mRNA levels were detected by qPCR assay at 36 h and 48 h after 2/3 PH. Data were expressed as mean ± SEM, *n* = 4–5, *P < 0.05, **P < 0.01, WT vs MANF^Hep−/−^.

Cyclin D1 is an important target of activated β-catenin [29–31]. To investigate whether MANF knockout affects β-catenin expression, firstly we compared the level of β-catenin in the liver tissues of sham group and the operated mice at 6 h after 2/3 PH in both WT and MANF^Hep−/−^ mice. We found that there was no significantly different between them (Fig. 5A). However, β-catenin expression significantly decreased at 48 h after 2/3 PH and gradually restored to normal levels after that in MANF^Hep−/−^ mice (Fig. 5C, D), while little change in β-catenin levels was observed in WT mice (Fig. 5B, D). This finding was confirmed at 48 h after 2/3 PH, the result showed that the levels of PCNA and β-catenin were simultaneously decreased in MANF^Hep−/−^ mice (Fig. 5E, F). The results of immunohistochemistry and qPCR were consistent with immunoblotting (Fig. 5G–I). More interestingly, we also found that β-catenin was primarily localized in the cytoplasm and nucleus in WT mice, whereas in MANF^Hep−/−^ mice, it was mainly distributed in the cell membrane (Fig. 5G). Moreover, the nuclear translocation of β-catenin was reduced in MANF^Hep−/−^ mice, compared with WT mice (Fig. 5G, indicated by arrows, Fig. 7H). Notably, the mRNA levels of β-catenin target genes were significantly down-regulated at 48 h in MANF^Hep−/−^ mice, compared with WT mice (Fig. 4F, Fig. 5J). These findings suggest that β-catenin plays an important role in MANF-mediated hepatocyte proliferation.

### MANF regulates the Wnt/β-catenin pathway after 2/3 PH

**Fig. 6:**
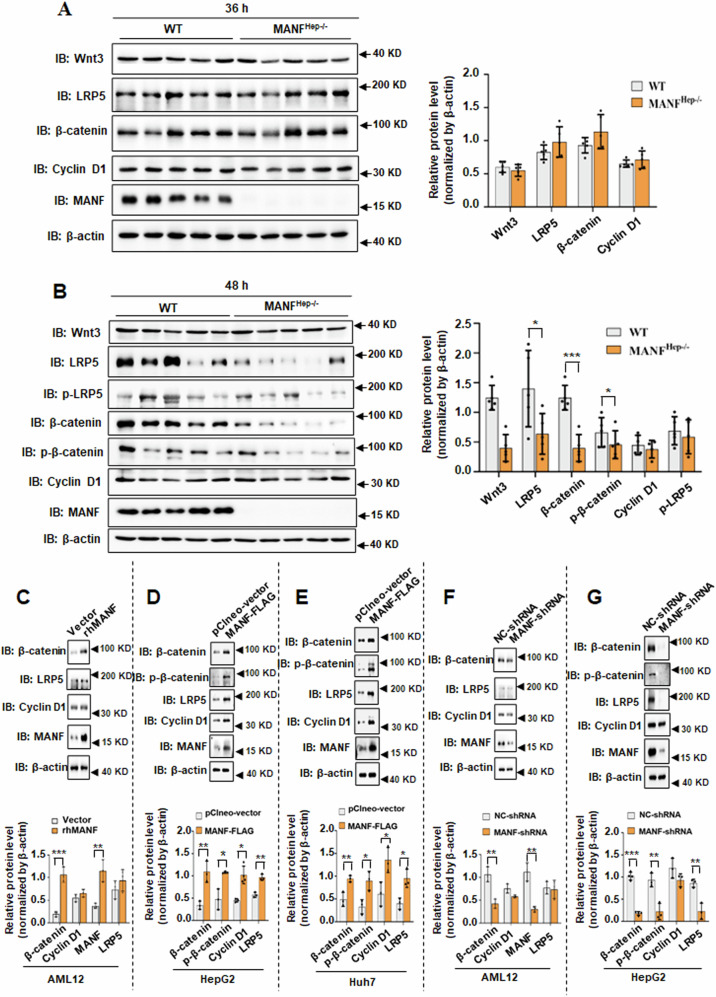
MANF activates the Wnt/β-catenin pathway after 2/3 PH. The protein levels of the Wnt target genes were detected at 36 h (**A**) or 48 h (**B**) after 2/3 PH by immunoblotting. Data were expressed as mean ± SEM, *n* = 5, *P < 0.05, ***P < 0.001, WT *vs* MANF^Hep−/−^. The protein levels of the Wnt target genes were detected in MANF-overexpressing or MANF-knocked down AML12 (**C**, **F**), HepG2 (**D**, **G**), and Huh7 (**E**) cells. Data were expressed as mean ± SEM, *n* = 3, *P < 0.05, **P < 0.01, ***P < 0.001, pClneo-vector vs MANF-FLAG, NC-shRNA *vs* MANF-shRNA.

β-catenin has been shown to play three significant roles in liver physiology [32], two of them are associated with cell proliferation. One is that β-catenin participates in the Wnt/β-catenin pathway, which is crucial for activating genes that are necessary for liver growth and regeneration [33–35]. Another is that β-catenin interacts with c-Met to form the c-Met/β-catenin complex, which induces hepatocyte proliferation [36, 37]. To investigate which role of MANF involved in liver, we firstly detected the target genes of Wnt/β-catenin pathway. The immunoblotting results showed no significant difference in the protein levels of the Wnt target genes indicated in Fig. 6A at 36 h between WT and MANF^Hep−/−^ mice. Furthermore, the levels of β-catenin, phosphorylated β-catenin, and LRP5 were significantly decreased at 48 h after 2/3 PH in MANF^Hep−/−^ mice, compared with WT mice (Fig. 6B). We also found that β-catenin, phosphorylated β-catenin, Cyclin D1, and LRP5 protein levels were significantly increased in MANF-overexpressing cells (Fig. 6C–E), while reduced in MANF-knocked down cells (Fig. 6F, G), compared with the controls. To investigate the effect of HGF/c-Met/β-catenin on MANF-mediated liver regeneration, the expression of HGF and c-Met were detected after 2/3 PH. The results revealed no significant difference between WT and MANF^Hep−/−^ mice (Supplementary Fig. S4). These results suggest that MANF promotes liver regeneration via Wnt/β-catenin pathway.

### MANF interacts with β-catenin and promotes its nuclear translocation

**Fig. 7:**
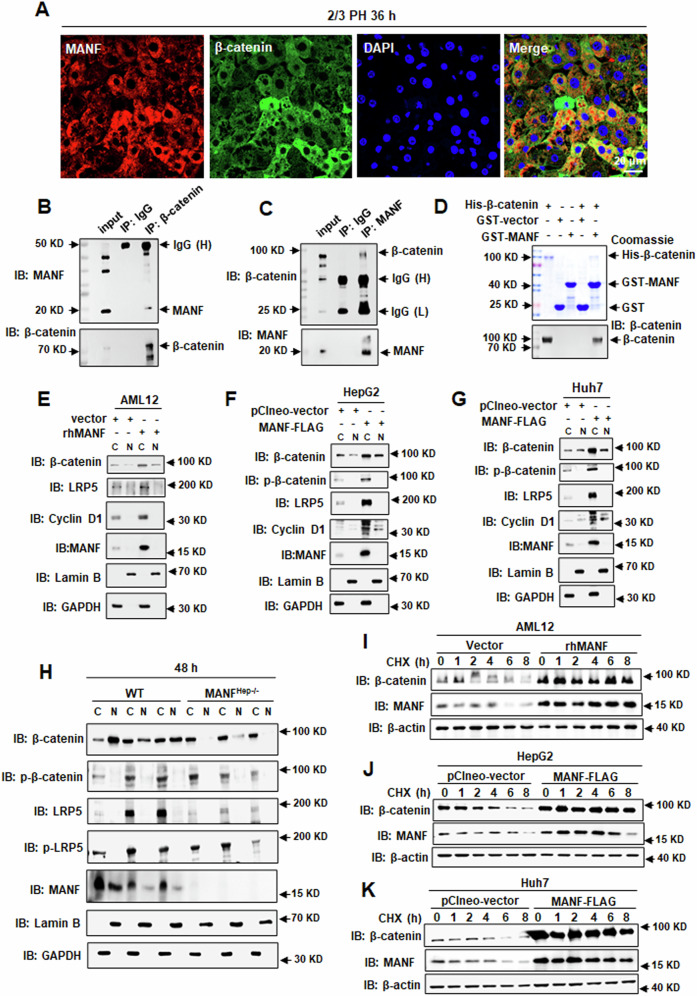
MANF interacts with β-catenin and promotes its nuclear translocation. **A** Immunofluorescence shows the co-localization of MANF (red) and β-catenin (green) in the liver tissues after 2/3 PH. DAPI was used to stain the nuclei (blue). Scale bar = 20 μm. **B**, **C** The interaction of MANF and β-catenin was detected by Co-IP in the liver tissues after 2/3 PH. The tissues were lysed for co-IP assay and precipitated by using anti-β-catenin (**B**) or anti-MANF (**C**) antibody. The isotype IgG was used as a negative control. The bound proteins were blotted by using the antibodies as indicated. IgG (**H**) and IgG (**L**) represent the heavy chain and light chain of IgG, respectively. **D** The interaction of MANF and β-catenin was detected by GST pull-down assay. GST-MANF and His-β-catenin fusion proteins were purified. GST protein was used as a negative control. The proteins were detected by Coomassie blue staining and immunoblotting, respectively. **E** AML12 cells were transfected with rhMANF or vector control. GAPDH and Lamin B were used as cytoplasm and nucleus markers, respectively. C: cytoplasm; N: nucleus. HepG2 (**F**) and Huh7 (**G**) cells were transfected with MANF-FLAG or corresponding control plasmid. GAPDH and Lamin B were used as cytoplasm and nucleus markers, respectively. C: cytoplasm; N: nucleus. **H** Nuclear and cytoplasmic proteins were extracted from liver tissues of WT and MANF^Hep−/−^ mice at 48 h after 2/3 PH. GAPDH and Lamin B were used as cytoplasm and nucleus markers, respectively. C: cytoplasm; N: nucleus. **I** The protein level of β-catenin was detected from AML12 cells treated with cycloheximide (CHX) for different time in the presence or absence of rhMANF. **J**, **K** The protein level of β-catenin was detected from HepG2 (J) and Huh7 (K) cells treated with cycloheximide (CHX) for different time in the presence or absence of MANF-FLAG.

To further investigate how MANF regulates Wnt/β-catenin signaling, we firstly observed the interaction of MANF and β-catenin. The immunofluorescence staining results showed that MANF co-localized with β-catenin (Fig. 7A, Supplementary Fig. S5A). Results of Co-IP verified that MANF was coimmunoprecipitated with β-catenin in the liver tissues after 2/3 PH either using anti-β-catenin (Fig. 7B) or anti-MANF antibody (Fig. 7C). GST pull-down assay showed that MANF physically interacts with β-catenin (Fig. 7D). To further understand the molecular mechanism underlying the interaction between MANF and β-catenin, we observed the effect of MANF on the subcellular localization. The results showed that overexpression of MANF in cells led to the increase in total level of β-catenin, as well as nuclear β-catenin (Fig. 7E–G, Supplementary Fig. S5B–D). Furthermore, the nuclear localization of β-catenin was decreased in MANF^Hep−/−^ mice, compared with WT mice (Fig. 7H, Supplementary Fig. S5E), indicating a role of MANF in regulating the subcellular localization of β-catenin. We also found that the level of phosphorylated β-catenin was increased in cytosol after MANF overexpression (Fig. 7F, G). To assess the effect of MANF on β-catenin stability, we performed a CHX chase assay on hepatoma cells overexpressing MANF. Our data showed that MANF overexpression markedly stabilized β-catenin (Fig. 7I–K, Supplementary Fig. S5F–H), suggesting that MANF may inhibit β-catenin degradation.

### MANF interacts with LRP5 after 2/3 PH

**Fig. 8:**
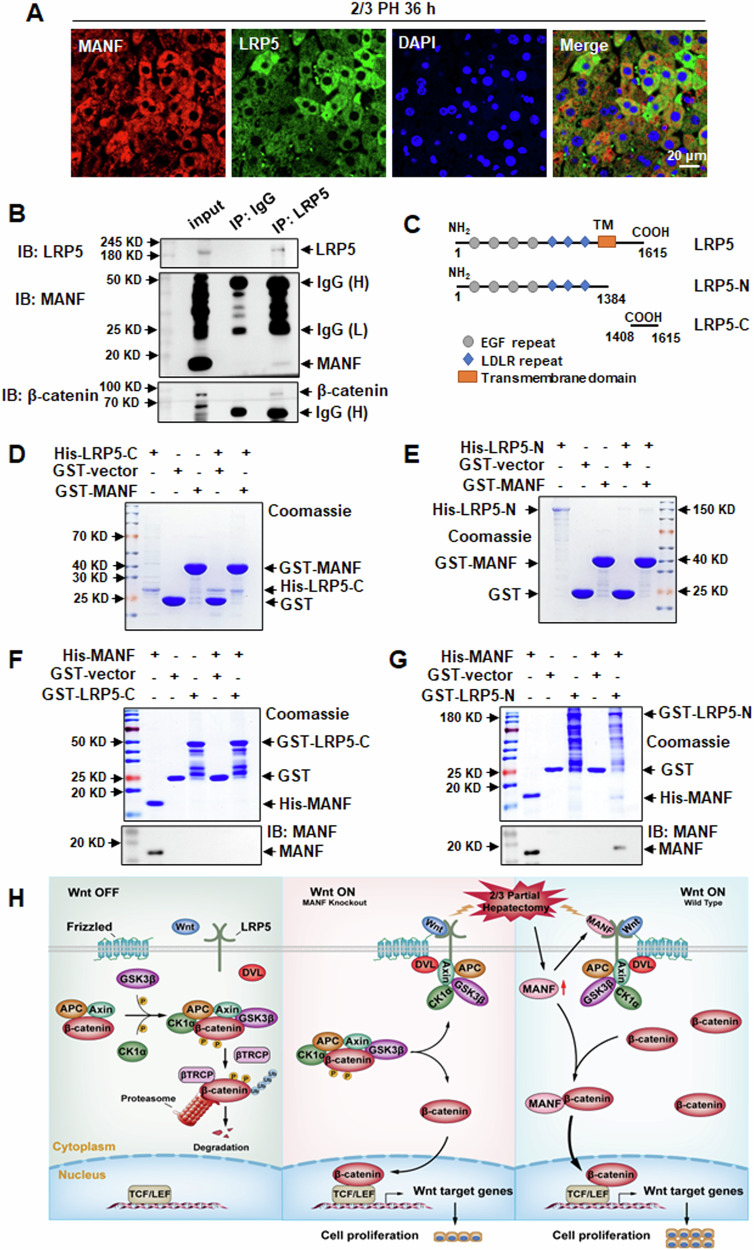
MANF interacts with LRP5. **A** Immunofluorescence shows the co-localization of LRP5 (green) and MANF (red) in the liver tissues after 2/3 PH. DAPI was used to stain the nuclei (blue). Scale bar = 20 μm. **B** The interaction of MANF and LRP5 was detected by Co-IP in the liver tissues after 2/3 PH. The tissues were lysed for Co-IP assay and precipitated by using anti-LRP5 antibody. The isotype IgG was used as a negative control. The bound proteins were blotted by using the antibodies as indicated. IgG (H) and IgG (L) represent the heavy chain and light chain of IgG, respectively. **C** Schematic representation of LRP5’s fragments. Two truncated forms of LRP5 include LRP5-N and LRP5-C. Numbers indicate the amino acid position. TM: transmembrane domain. **D–G** The interaction of MANF and LRP5 was detected by GST pull-down assay. GST-MANF, His-LRP5-C, His-LRP5-N, His-MANF, GST-LRP5-C, and GST-LRP5-N fusion proteins were purified. GST protein was used as a negative control. The proteins were detected by Coomassie blue staining and immunoblotting, respectively. **H** Schematic diagram of MANF in regulating Wnt/β-catenin pathway.

To better understand the molecular mechanism of MANF in liver regeneration, we screened the potential interacting proteins of MANF by using Co-IP plus mass spectrometry analysis. Low-density lipoprotein receptor-related protein 5 (LRP5), a coreceptor of Wnt/β-catenin pathway, was identified as in the complex immunoprecipitated with MANF antibody (Supplementary Fig. S6A, B). The immunofluorescence staining results showed that MANF co-localized with LRP5 (Fig. 8A, Supplementary Fig. S6C). Co-IP analysis was conducted with an antibody against LRP5 using mice liver lysates after 2/3 PH. The result showed that MANF interacted with LRP5 (Fig. 8B). Interestingly, β-catenin was also found in the pull-down protein complex of LRP5 (Fig. 8B), suggesting that LRP5 may be associated with β-catenin via MANF. MANF is also a secreted protein [10, 11, 38]. To elucidate where MANF interacts with LRP5, extracellularly or intracellularly, we constructed the truncates of LRP5 according to its extracellular domain (LRP5-N) and the intracellular domain (LRP5-C) (Fig. 8C). The results of GST pull-down assay revealed that only the extracellular segment of LRP5 (LRP5-N) interacted with MANF (Fig. 8D–G). These findings suggest that MANF physically interacts with the extracellular segment of LRP5.

## Discussion

Liver regeneration following surgical resection is a complex process that includes various cytokines and growth factor-mediated pathways. MANF was originally identified as a member of a new NTFs family, its functions have been extensively studied, particularly in brain and liver disorders. Evidence suggests that MANF rescues neuronal loss in several neurological disorders, such as Alzheimer’s disease, Parkinson’s disease, and cerebral ischemia [13, 14, 16]. Previous studies in our group have demonstrated that MANF plays a protective role in liver injuries induced by drugs [25, 26], alcohol [27], and ischemia reperfusion [24]. Here, we present the first evidence of up-regulation of MANF expression in liver tissues after 2/3 PH, and demonstrated a novel role of hepatocyte-derived MANF in mice liver regeneration induced by 2/3 PH. We found that MANF expression was up-regulated in a time-dependent manner in the liver tissues after 2/3 PH and MANF knockout delayed hepatocytes proliferation, indicating MANF is involved in liver regeneration. The rate of liver mass recovery was significantly decreased, DNA replication and cell cycle progression were delayed in MANF deficiency by 12–24 h after 2/3 PH. Cyclin D1 is a key regulator ushering hepatocytes into the S phase. Its activation and translocation to the nucleus lead to DNA synthesis in hepatocytes, which is the key event initiating cell proliferation [39]. We found that MANF knockout weakens the induction of Cyclin D1. The impairing of Cyclin D1 induction is likely responsible for the delay in DNA synthesis in MANF knockout mice.

β-catenin plays important roles in cell proliferation, differentiation, and stem cell maintenance in a wide variety of tissues [37, 40]. There are three major β-catenin- related pathways in liver physiology, including Wnt/β-catenin pathway, E-cadherin/β-catenin, and c-Met/β-catenin complex. Wnt/β-catenin pathway and c-Met/β-catenin complex were closely associated with hepatocyte proliferation [32, 33, 37]. Previous studies have reported that liver-specific loss of β-catenin lead to a 12–24 h delay of both DNA replication and cell cycle progression, a decrease in the level of Cyclin D1 as well in mice [41, 42]. In this study, we observed β-catenin level was significantly attenuated in MANF deficient mice at 48 h after 2/3 PH. MANF overexpression increased β-catenin level, whereas MANF knockdown decreased its expression. In addition, the target genes of Wnt/β-catenin pathway were significantly decreased at 48 h in MANF deficient mice. However, we did not find the change in HGF/c-Met level in MANF deficient mice after 2/3 PH. Therefore, the delay of cell proliferation caused by MANF deficiency may be associated with Wnt/β-catenin pathway.

The Wnt/β-catenin pathway is inactive in normal unstimulated mature cells, this steady-state condition is ensured by the absence of Wnt ligands and the degradation of β-catenin (Wnt-OFF state) (Fig. 8H, left). Without a Wnt signal, cytosolic β‐catenin is actively phosphorylated by a destruction complex comprising adenomatous polyposis coli (APC), Axin, glycogen synthase kinase 3β (GSK3β), and casein kinase 1α (CK1α). β-catenin is serially phosphorylated at serine-45 by CK1α and at serine-33, serine-37 and threonine-41 by GSK3β. Phosphorylated β-catenin is subsequently recognized by β-transducin repeat-containing protein (βTRCP), an E3 ubiquitin ligase, which mediates its ubiquitylation and promotes its degradation by the proteasome [43, 44]. As a result, cytoplasmic β-catenin is maintained at a low level. By contrast, in the activated (Wnt-ON) state, β-catenin functions as a central effector of the Wnt signaling cascade. Binding of active Wnt to the Frizzled receptor and LRP5/6 co-receptors in an autocrine or paracrine manner. This coreceptor complex triggers activation of the Wnt/β-catenin pathway. Disheveled (DVL) is recruited to the Frizzled receptor. The LRP5/6 receptors bind to Axin. DVL and Axin are recruited to this membrane‐anchored protein complex, leading to instability of the β-catenin destruction complex and failure to degrade β-catenin. The cytoplasmic β-catenin was increased and translocated into the nucleus interacting with T cell-specific transcription factor/lymphoid enhancer-binding factor (TCF/LEF) to initiate the transcription of Wnt target genes, which plays a critical role in the regulation of diverse cell behaviors, including cell differentiation, proliferation, and migration [45, 46]. In this study, we found β-catenin was primarily distributed in the cytoplasm and nucleus in WT mice, whereas it was mainly localized in cell membrane in MANF deficient mice after 2/3 PH, suggesting MANF knockout decreases the nuclear translocation of β-catenin. Consistently, we found that MANF overexpression increased the total and nuclear β-catenin levels. Meanwhile, we have demonstrated that MANF physically interacts with β-catenin, which may inhibit its degradation by proteasome and stabilize its level in cytosol. The high level of cytosolic β-catenin is required for its nuclear translocation, which further enhances the Wnt/β-catenin signaling.

LRP5 is a member of the low-density lipoprotein receptors (LDLR) family. As a co-receptor of Wnt/β-catenin ligands and key component of its receptor complex, LRP5 is necessary for Wnt/β-catenin signaling [47]. Here, we identified LRP5 as an interacting protein of MANF, and the interacting domain is within the extracellular segment of LRP5. That means that extracellular MANF may bind to LRP5. The next question is where the extracellular MANF comes from? Just as we mentioned above, MANF is a secreting protein. It was up-regulated by 2/3 PH and secreted into the outside of hepatocytes. The secreted MANF with high level then binds to extracellular segment of LRP5. Therefore, MANF can activate the Wnt/β-catenin signaling at the upstream of this signal pathway as a co-factor of LRP5. We also found MANF overexpression increased the total level of LRP5, which may enhance the impact of LRP5 on Wnt/β-catenin signaling. However, regarding how LPR5 was up-regulated by MANF and the details about the interaction of MANF and LRP5 need to be further explored.

In summary, MANF promotes hepatocyte proliferation induced by 2/3 PH via activating Wnt/β-catenin pathway. The proposed mechanistic scheme is summarized in Fig. 8H. There are two potential targets of MANF in Wnt/β-catenin pathway. One is LPR5, and another is β-catenin. Upon 2/3 PH, upregulation of MANF enhances the binding of MANF to the extracellular segment of LRP5, which synergistically acts with Wnt to activate the Wnt/β-catenin pathway. On the other hand, upregulation of MANF stabilizes cytosolic β-catenin to promote its nuclear transport and enhance the Wnt/β-catenin signaling. These findings help us to understand the mechanisms of liver regeneration and shed light on the potential application of MANF.

## Materials and methods

### 2/3 partial hepatectomy model

In this study, we used the hepatectomy method established by Claudia Mitchell and Holger Willenbring in 2008 [7], and liver specimens were collected at different time points (sham, 6, 12, 24, 36, 48, 72, and 168 h) after 2/3 PH. All surgeries were performed between 08:00 AM and 12:00 AM. Blood collection by retro-orbital puncture was used to examine serum aspartate aminotransferase (AST), alanine aminotransferase (ALT), and the liver weight to body weight ratio was measured after 2/3 PH.

### Animals

All the animal experiments were approved by the Animal Experimental Committee of Anhui Medical University. MANF^flox/flox^ mice bearing loxP sites flanking exons 3 of the *manf* gene on the C57BL/6 background were kindly provided by Prof. Jia Luo of Kentucky University in the USA. MANF^flox/flox^ mice were cross-bred with albumin-cre mice to specifically knockout *manf* gene in hepatocytes (MANF^flox/flox^ Alb-Cre-T, MANF^Hep−/−^). The mating and reproduction of MANF^Hep−/−^ was commissioned by GemPharmatech Co., Ltd. (Nanjing, China). The genotyping of MANF^Hep−/−^ mice was performed by PCR on tail DNA using primer pairs. 12-week-old C57BL/6 mice and MANF^Hep−/−^ mice were used in the study. His-tagged recombinant human MANF (rhMANF, 1 mg/kg) was administrated to WT mice by tail vein. Mice were kept on a 12-h light/dark cycle conditions with free access to food and water.

### Immunohistochemistry

For histological analyses, liver tissues were fixed in 10% neutral formaldehyde, dehydrated in a graded alcohol series, and embedded in paraffin. Paraffin-embedded liver samples were cut at 4-μm-thick sections. For immunohistochemistry staining, all proteins were visualized using the avidin-biotin-peroxidase complex technique and counterstained with hematoxylin. The number of positive cells per high-power field was counted in 6 randomly selected fields per liver (at least 5 mice/group/time point). We expressed all values as mean ± SEM. The following antibodies were used during this study: MANF (abcam, ab126321), Ki67 (abcam, ab15580), PCNA (Cell Signaling Technology, 13110), CyclinD1 (Cell Signaling Technology, 2978), β-catenin (Cell Signaling Technology, 37447), LRP5 (Cell Signaling Technology, 5731), LRP5 (Santacruz, sc-390267), phosphorylated LRP5 (Affinity, AF4345), phosphorylated β-catenin (Affinity, DF2989), HGF (Affinity, DF6326), c-Met (Affinity, AF6128), Wnt3 (Affinity, DF13430), Lamin B (Abways, AB0054), GAPDH (Elabscience, E-AB-20032), β-actin (Affinity, AF7018), and His (proteintech, 66005-1-Ig).

### Quantitative real-time polymerase chain reaction (qPCR)

All the RNA samples were extracted using Trizol reagent (TaKaRa) according to the manufacturer’s protocol and RNA concentration was measured by spectrophotometer. Each test was repeated three times and the expression of relative genes was determined based on the 2^−△△Ct^ method. Expression of liver genes was compared with the expression level of β-actin. The primer sequence of qPCR assay was listed in Table 1.

**Table 1:** Gene-specific primer sequences used for qPCR.

Gene	Source	Forward	Reverse
MANF	Human	TCACATTYTCACCAGCCACT	ATCTGGCTGTCYTTCTTCTTMA
PCNA	Mouse	TACAGCTTACTCTGCGCTCC	TGTCTGCATTATCTTCAGCCCT
Ki67	Mouse	ATCATTGACCGCTCCTTTAGGT	GCTCGCCTTGATGGTTCCT
CyclinD1	Mouse	AAGCATGCACAGACCTTTGTGG	TTCAGGCCTTGCATCGCAGC
HGF	Mouse	AACAGGGGCTTTACGTTCACT	CGTCCCTTTATAGCTGCCTCC
c-Met LPR5	Mouse Mouse	ACAGTGGCATGTCAACATCGCT AGACCAATAACAACGACGTG	GCTCGGTAGTCTACAGATTC TTCTTGCCCATCCAGTC
β-catenin	Mouse	GCTGATTTGATGGAGTTGGAC	AGGAGCTGTGGTAGTGGCACCAG
β-actin	Mouse	CTCCATCCTGGCCTCGCTGT	TCGGACACATGAGCCATGAT

### Immunoblotting

Protein samples were extracted from liver homogenate using RIPA buffer (Beyotime) containing a mixture of protease inhibitors. 12% polyacrylamide gel was separated by SDS-PAGE and transferred onto PVDF membrane (Invitrogen) by electrophoresis. The membranes were blocked with 5% nonfat milk in Tris-buffered saline for 1 h at room temperature and then incubated overnight with specific primary antibodies. The immunoblotted protein signals were detected using an ECL enhanced chemiluminescence system with chemiluminescent substrates (Advansta). β-actin was used as a protein-loading control.

### Cell culture and transfection

AML12, HepG2, and Huh7 cells were seeded in 24-well plate and cultured overnight in saturated incubator at 37 °C and 5% CO_2_. When the cell density reached 80%, plasmids were transfected using lipofectamine plus reagent (Invitrogen) according to the manufacturer’s instructions. The primary hepatocytes were isolated from the liver tissues of MANF^Hep−/−^ mice using a two-step collagenase perfusion system (Sigma-Aldrich, C5138). The cells were plated on collagen-coated 6-well plate at a density of 2×10^5^ cells/well, and treated with HGF (MCE, HY-P73928, 100 ng/ml), and then recombinant human MANF (10 μg/mL) was added for 12 h.

### Co-immunoprecipitation

The protein A/G agarose beads (Thermo Fisher Scientific, 20421) was incubated with the primary antibody for 4 h. Liver tissues were homogenized with IP lysis buffer (Beyotime) containing protease inhibitor cocktail. The lysate was centrifuged at 12,000 × *g* at 4 °C for 10 min, and 8% of the supernatant was used for input. The protein A/G-antibody-antigen complex was placed overnight in a rotating incubator at 4 °C. Then, the samples were washed with lysis buffer for three times. After the last washing, SDS loading buffer was added, the immunoprecipitants were boiled and resolved by electrophoresis for immunoblotting analysis.

### Glutathione S-transferase protein pull-down assay

The following plasmids were used during this study: GST-MANF (pGEX 6P-1), His-MANF (pET-28a), His-LRP5-N (pET-28a), His-LRP5-C (pET-28a), His-β-catenin (pET-28a), GST-LRP5-N (pET-41a), and GST-LRP5-C (pET-41a). Expression vectors were transformed into Escherichia coli strain BL21 (DE3), and proteins were expressed by IPTG induction. GST-tag recombinant proteins were purified using the Glutathione Sepharose^TM^ 4B (GE Healthcare) according to manufacturer’s instructions. The GST-tag fusion protein purified and immobilized on glutathione Sepharose beads were incubated at 4 °C for 1 h. Then, purified His-tag protein was added for pull-down experiment, and the beads/protein complex was washed, and immunoblotting was performed.

### Cycloheximide chase assay

Cycloheximide (CHX) is a common protein synthesis inhibitor in eukaryotes. CHX chase assay was performed by using CHX (MedChemExpress, HY-12320). HepG2 and Huh7 cells were transfected with MANF-FLAG plasmid. Cells were treated with 100 μg/ml CHX at 36 h after transfection. Protein lysates were collected in 0, 1, 2, 4, 6, and 8 h after CHX treatment. Western blotting was performed as described above.

### Statistical analysis

All data were presented as mean ± standard error (SEM). A one-way ANOVA analysis of variance (ANOVA) or two-tailed Student’s *t* test was used for data analysis. Differences with values of *P* < 0.05 was considered statistically significant.

## Supplementary information

Supplementary information: Fig S1 to S7 for multiple supplementary figures

original data from immunoblotting

## Data Availability

The data analyzed during this study are included in this article and the supplemental data files. The data supporting the findings of this study are available from the corresponding author upon reasonable request.
