# Supplementary material for: MANF serves as a novel hepatocyte factor to promote liver regeneration after 2/3 partial hepatectomy via doubly targeting Wnt/β-catenin signaling
Source: Cell Death Dis. 2024 Sep 18;15(9):681. doi: 10.1038/s41419-024-07069-8 (PMC11408687; doi:10.1038/s41419-024-07069-8)
Supplement: Supplementary file 1 — Supplementary information: Fig S1 to S7 for multiple supplementary figures [file 41419_2024_7069_MOESM1_ESM.docx]

**Fig S1 to S7 for multiple supplementary figures:**


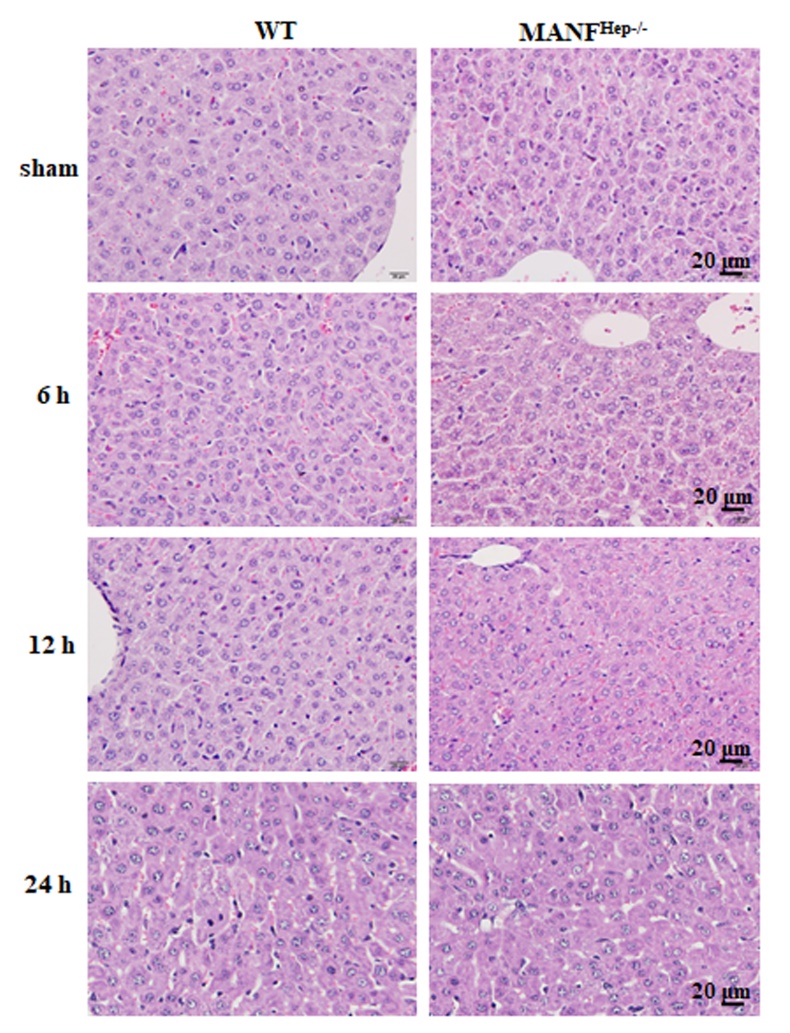


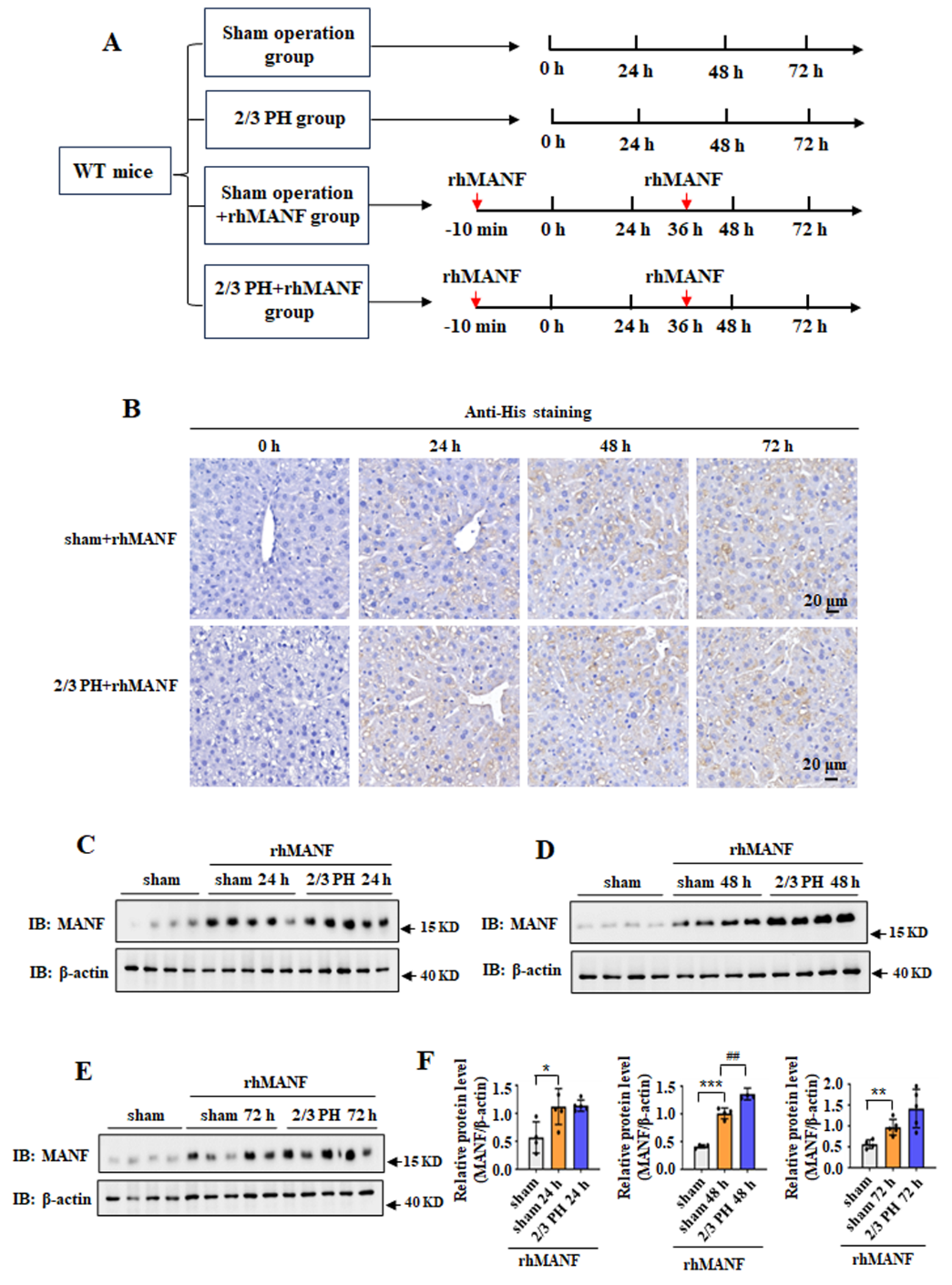


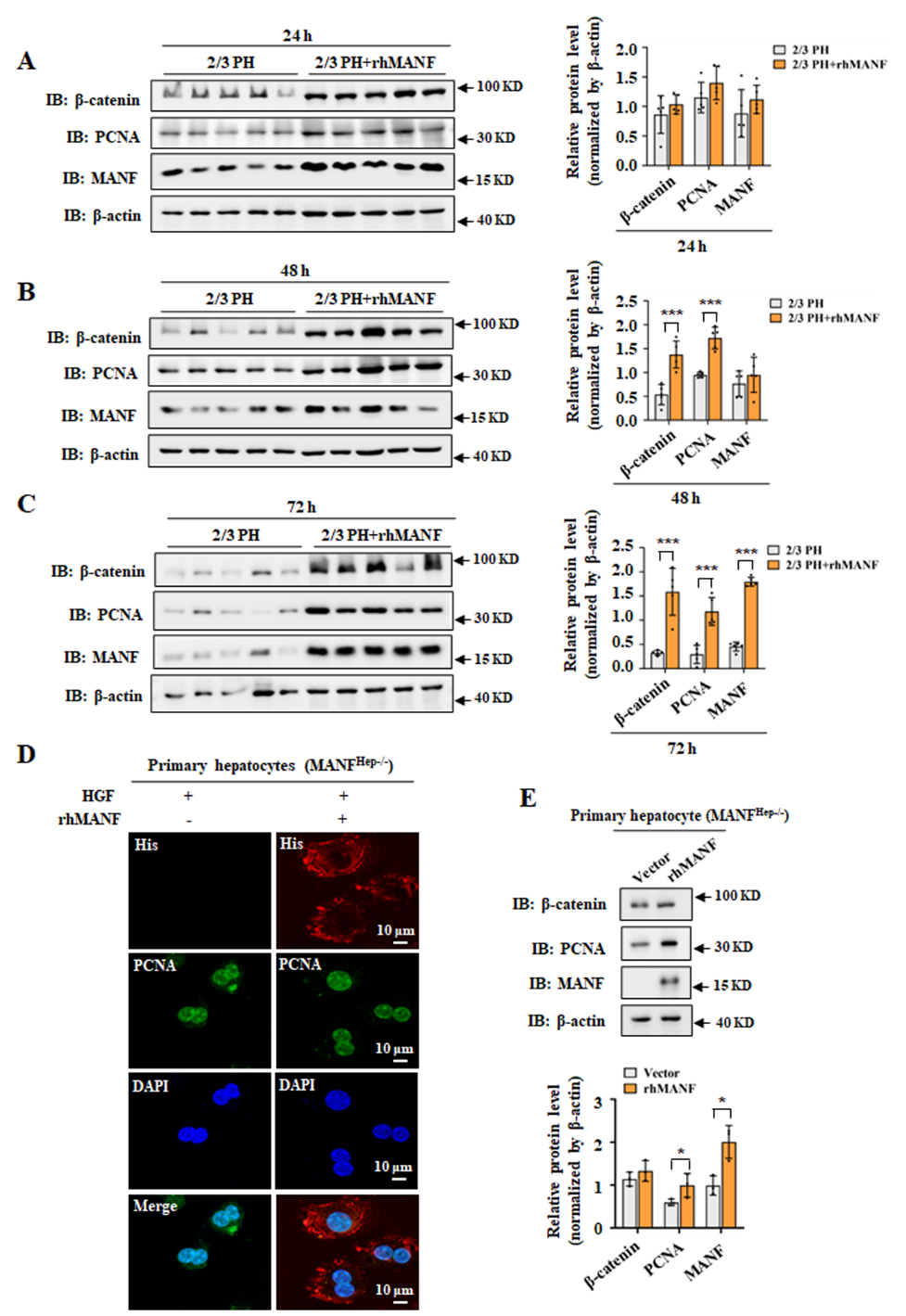


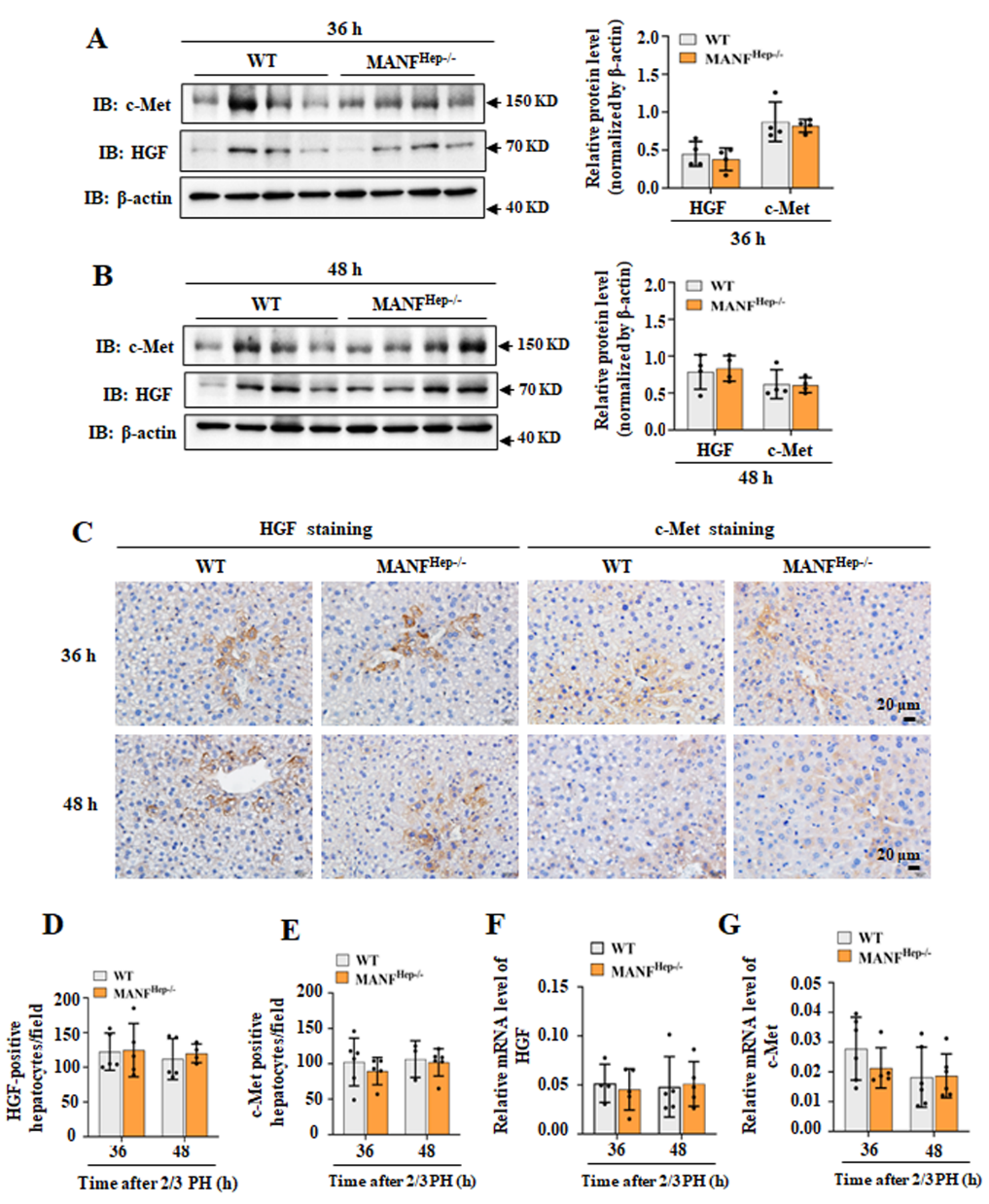


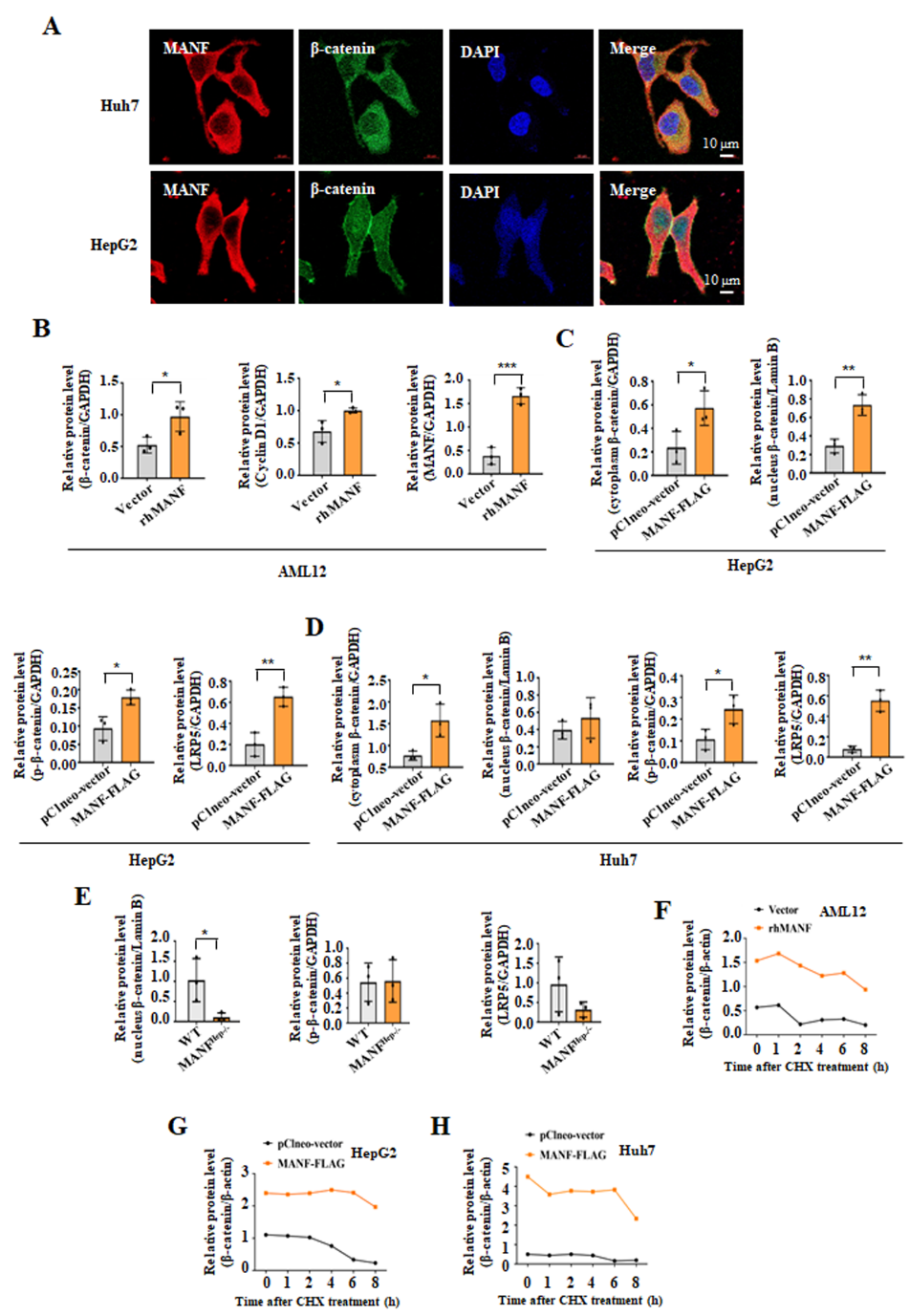


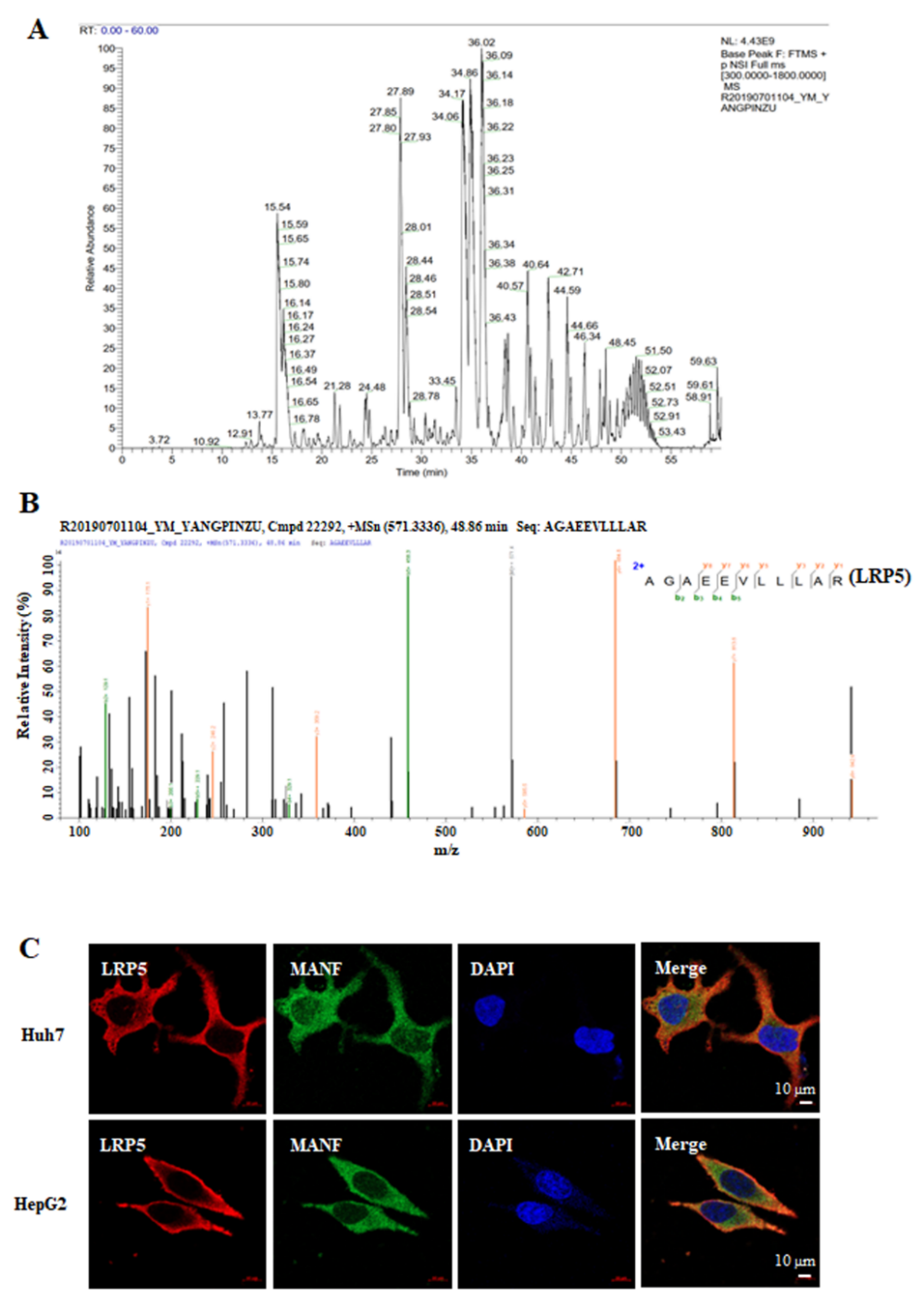


**Figure legends**

**Supplementary Fig. S1 No morphological differences between WT and MANF^Hep-/-^ mice after 2/3 PH**. Hematoxylin and eosin staining of the liver tissues in sham group and operation group at 6, 12, and 24 hours after 2/3 PH. Scale bar = 20 μm.

**Supplementary Fig. S2 The schedule of time points for 2/3 PH and rhMANF administration in mice. A** The schedule of time points for 2/3 PH and rhMANF administration in mice. **B** The presence of His-tagged rhMANF in the liver tissues was detected by using immunohistochemistry with anti-His antibody. **C-F** The level of MANF in the liver tissues at the indicated time points after 2/3 PH was detected by using immunoblotting with anti-MANF antibody after rhMANF administration. Data were expressed as mean ± SEM, n = 4-5, * *P* < 0.05, ** *P* < 0.01, *** *P* < 0.01, sham *vs* sham+rhMANF. ##*P* < 0.01, sham *vs* 2/3 PH+rhMANF.

**Supplementary Fig. S3 Recombinant human MANF (rhMANF) promotes hepatocyte proliferation** **after 2/3 PH. A-C** MANF, PCNA, and β-catenin protein level were detected by immunoblotting in WT mice liver tissues after 2/3 PH and rhMANF administration. Data were expressed as mean ± SEM, n = 5, *** *P* < 0.01, 2/3 PH *vs* 2/3 PH+rhMANF. **D** The His-tagged rhMANF protein were detected by using immunofluorescence with anti-His antibody in primary hepatocytes from MANF^Hep-/-^ mice. DAPI was used to stain the nuclei (blue). Scale bar = 10 μm. **E** MANF, PCNA, and β-catenin levels were detected by immunoblotting in primary hepatocytes from MANF^Hep-/-^ mice after rhMANF administration. Data were expressed as mean ± SEM, n = 3, * *P* < 0.05, vector *vs* rhMANF.

**Supplementary Fig. S4 MANF knockout does not affect the c-Met/β-catenin complex after 2/3 PH. A, B** HGF and c-Met protein levels were detected by immunoblotting at 36 hours (A) and 48 hours (B) after 2/3 PH. Data were expressed as mean ± SEM, n = 4, WT *vs* MANF^Hep-/-^. **C** HGF and c-Met levels were detected by immunohistochemical in WT and MANF^Hep-/-^ mice livers after 2/3 PH. Scale bar = 20 μm. **D, E** The quantitative data in panel C. Data were expressed as mean ± SEM, n = 5, WT *vs* MANF^Hep-/-^. **F, G** HGF and c-Met mRNA levels were detected by qPCR assay. Data were expressed as mean ± SEM, n = 5, WT *vs* MANF^Hep-/-^.

**Supplementary Fig. S5 MANF interacts with β-catenin. A** Immunofluorescence shows the co-localization of MANF (red) and β-catenin (green) in hepatoma cells. DAPI was used to stain the nuclei (blue). Scale bar = 10 μm. **B-D** The quantitative data in panel Fig. 7E-G. Data were expressed as mean ± SEM, n = 3, * *P* < 0.05, ** *P* < 0.01, pClneo-vector *vs* MANF-FLAG. **E** The quantitative data in panel Fig. 7H. Data were expressed as mean ± SEM, n = 3, * *P* < 0.05, WT *vs* MANF^Hep-/-^. **F-H** The quantitative data in panel Fig. 7I-K.

**Supplementary Fig. S6 Screening MANF interacting proteins by mass spectrometry. A** Proteins interacting with MANF were detected by mass spectrometry. **B** The peak map of LRP5 acquired from the mass spectrometry. **C** Immunofluorescence shows the co-localization of MANF (green) and LRP5 (red) in hepatoma cells. DAPI was used to stain the nuclei (blue). Scale bar = 10 μm.
