# Supplementary figures and images for: MANF serves as a novel hepatocyte factor to promote liver regeneration after 2/3 partial hepatectomy via doubly targeting Wnt/β-catenin signaling
Source: Cell Death Dis. 2024 Sep 18;15(9):681. doi: 10.1038/s41419-024-07069-8 (PMC11408687; doi:10.1038/s41419-024-07069-8)

**original data from immunoblotting:**


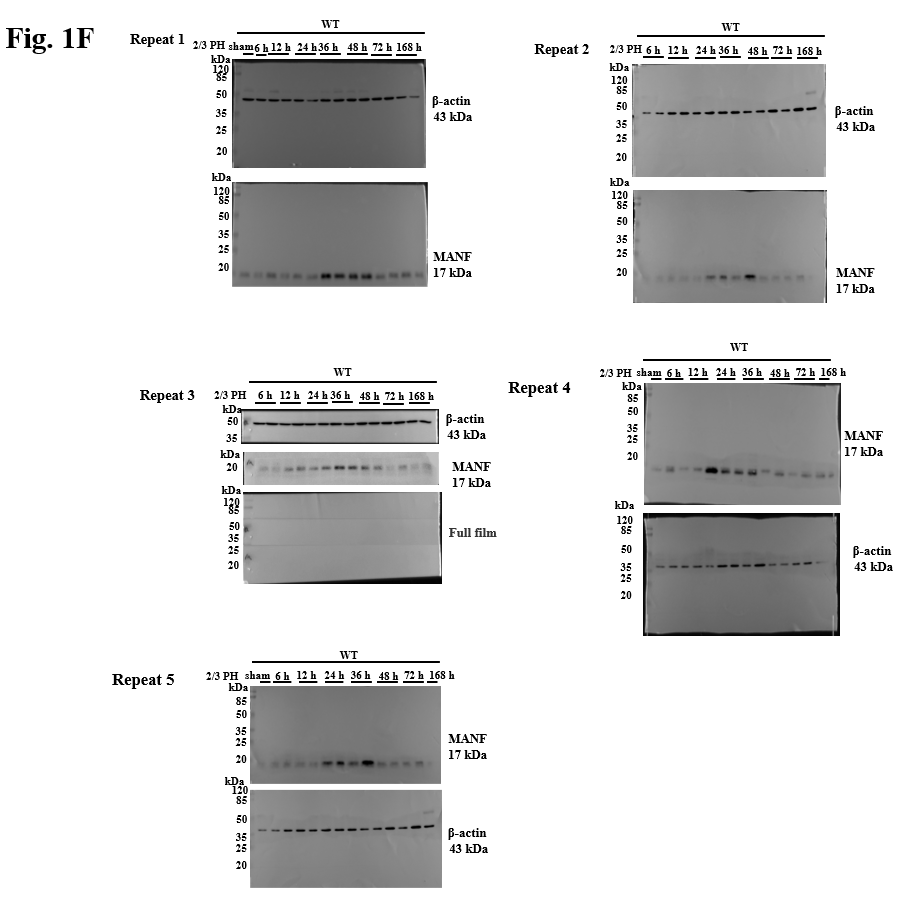


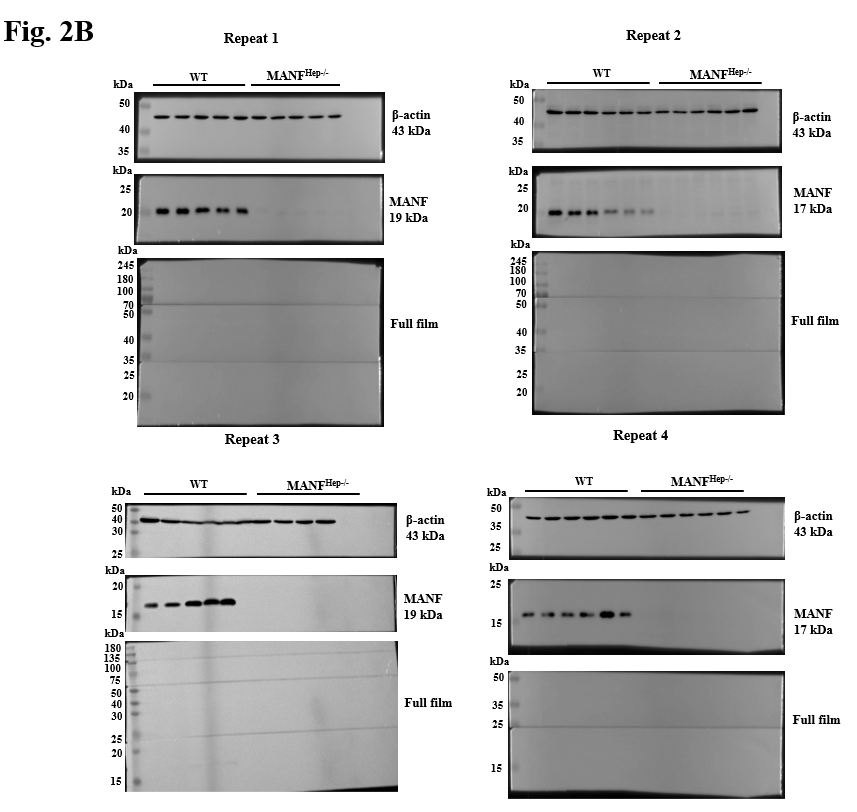


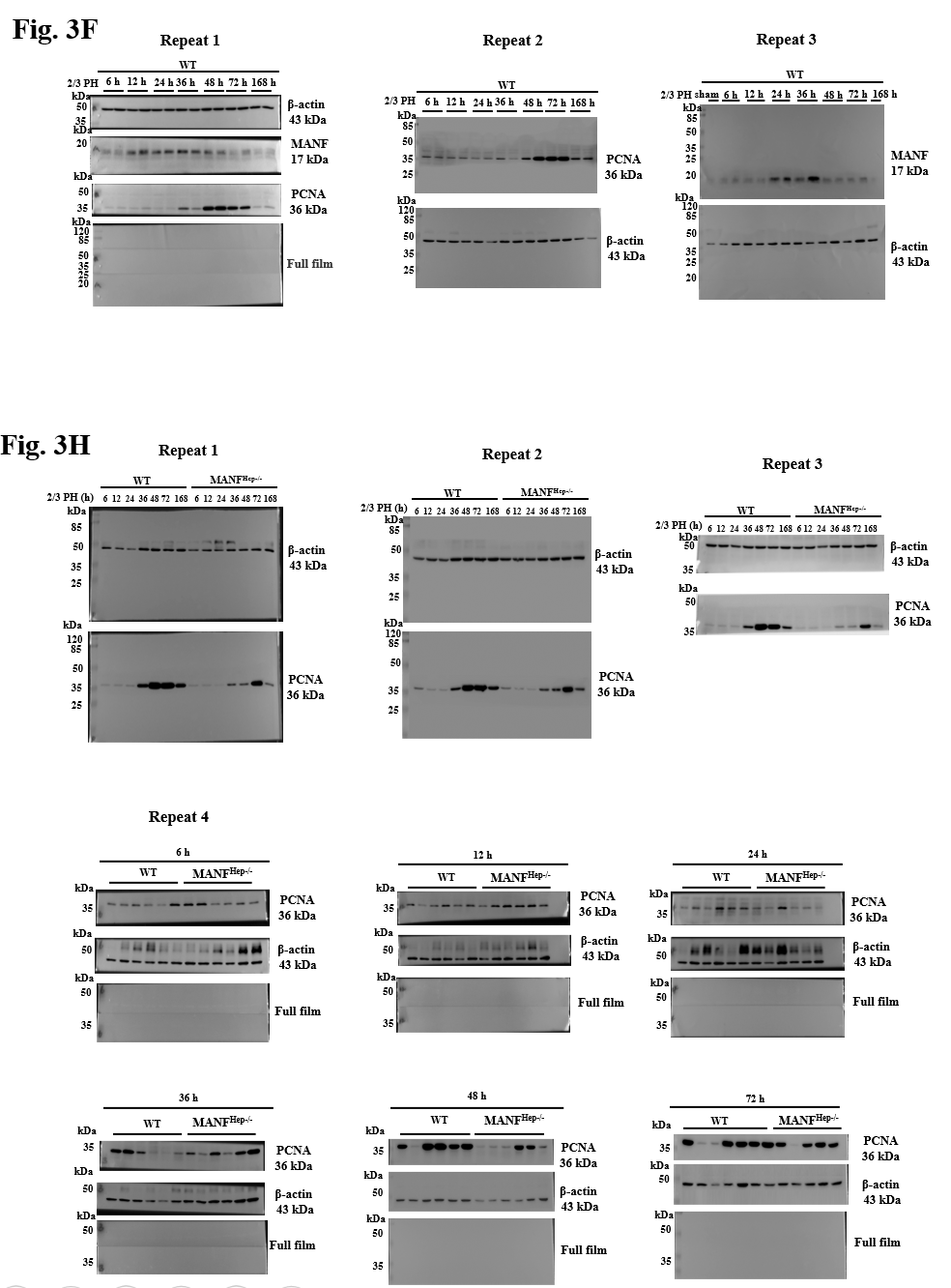


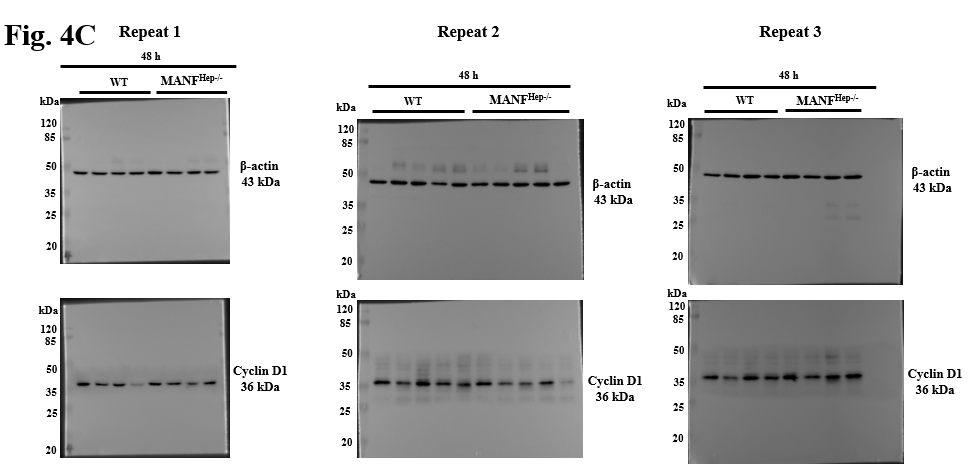


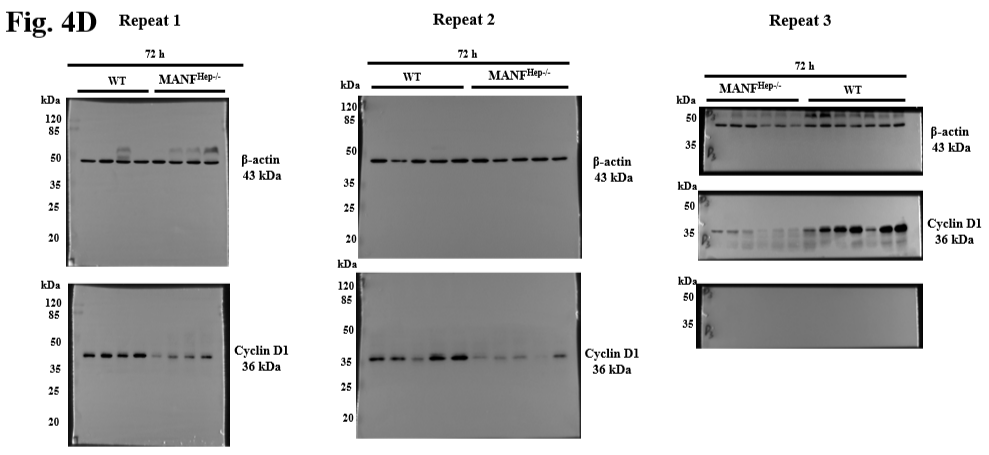


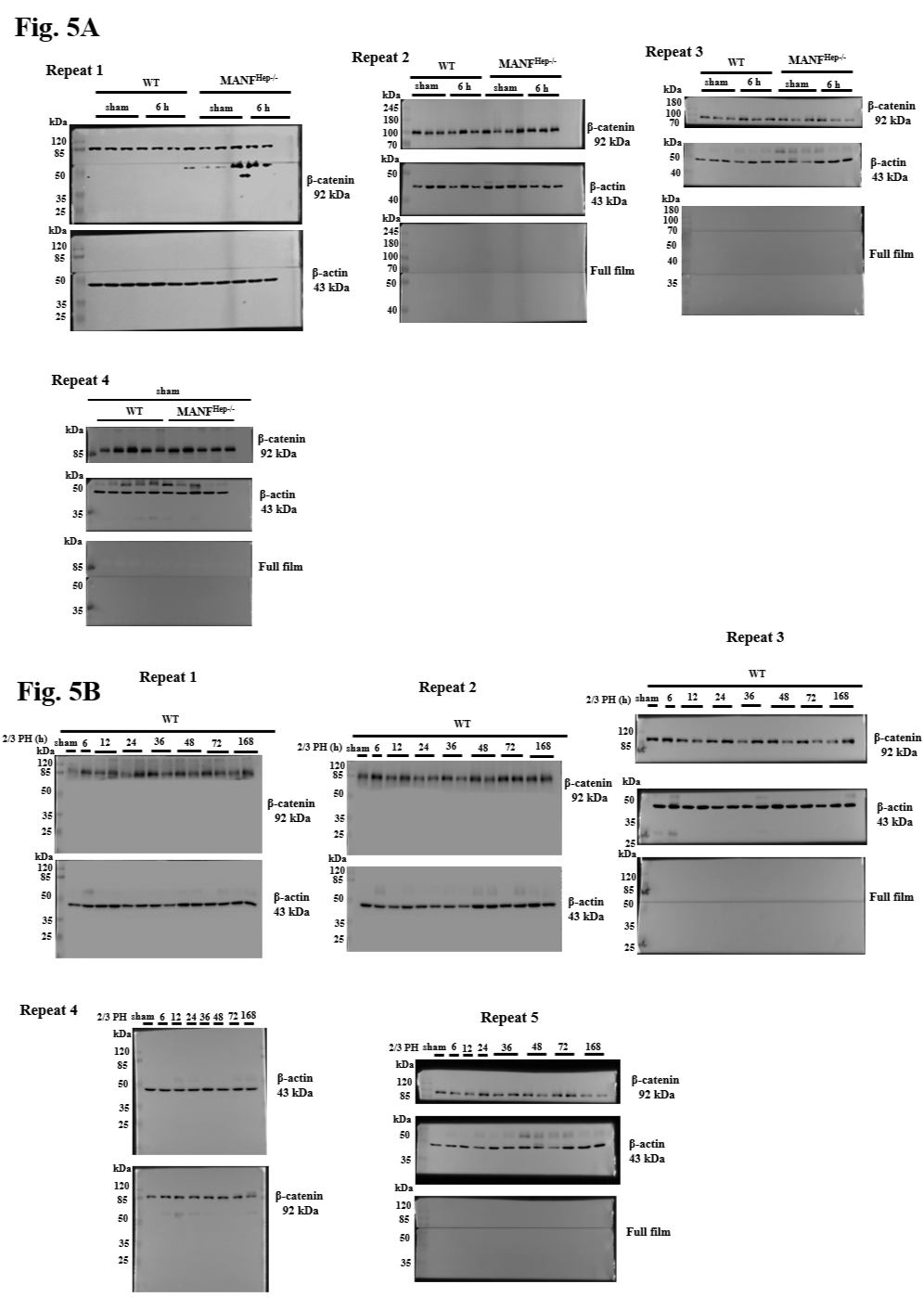


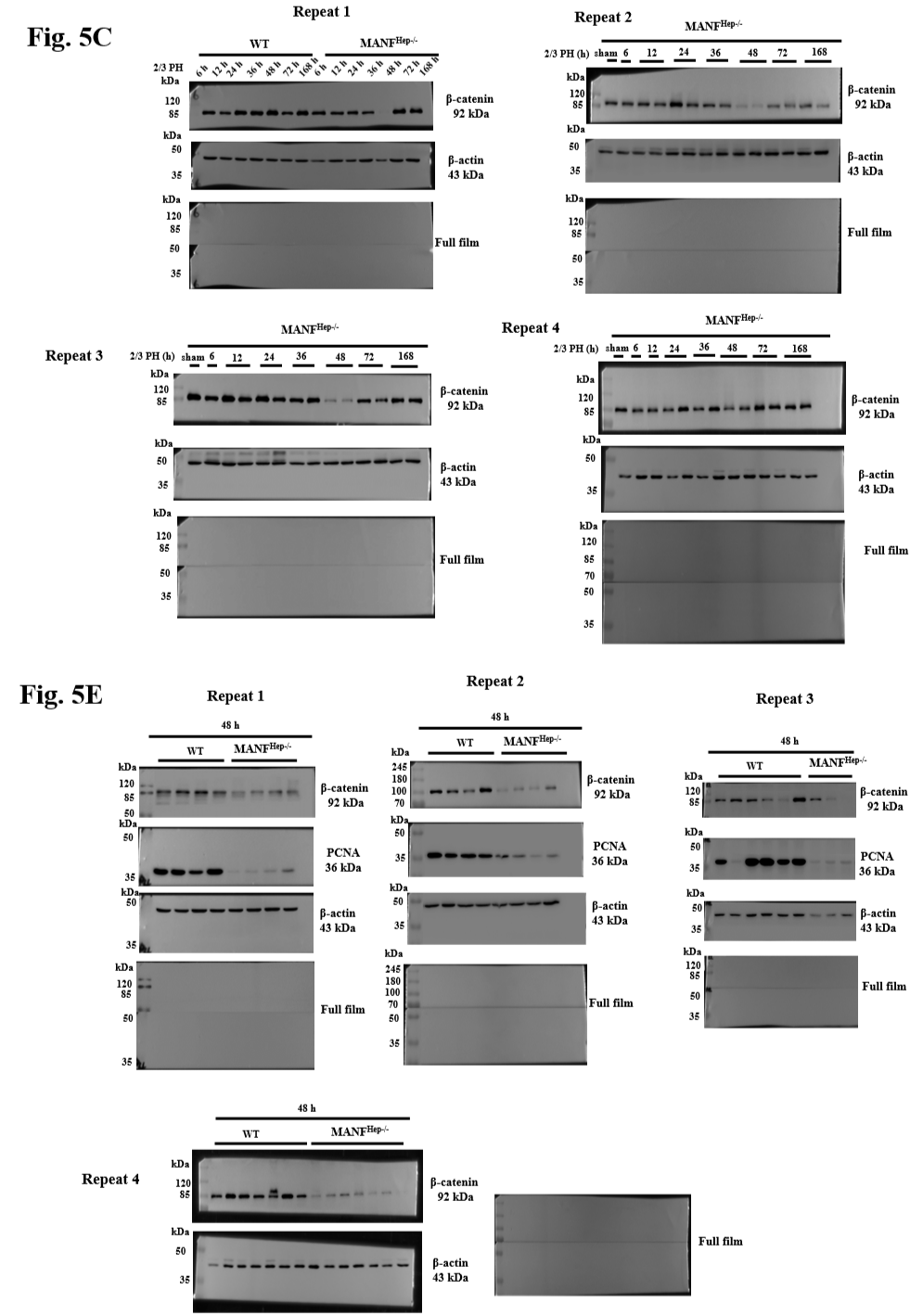


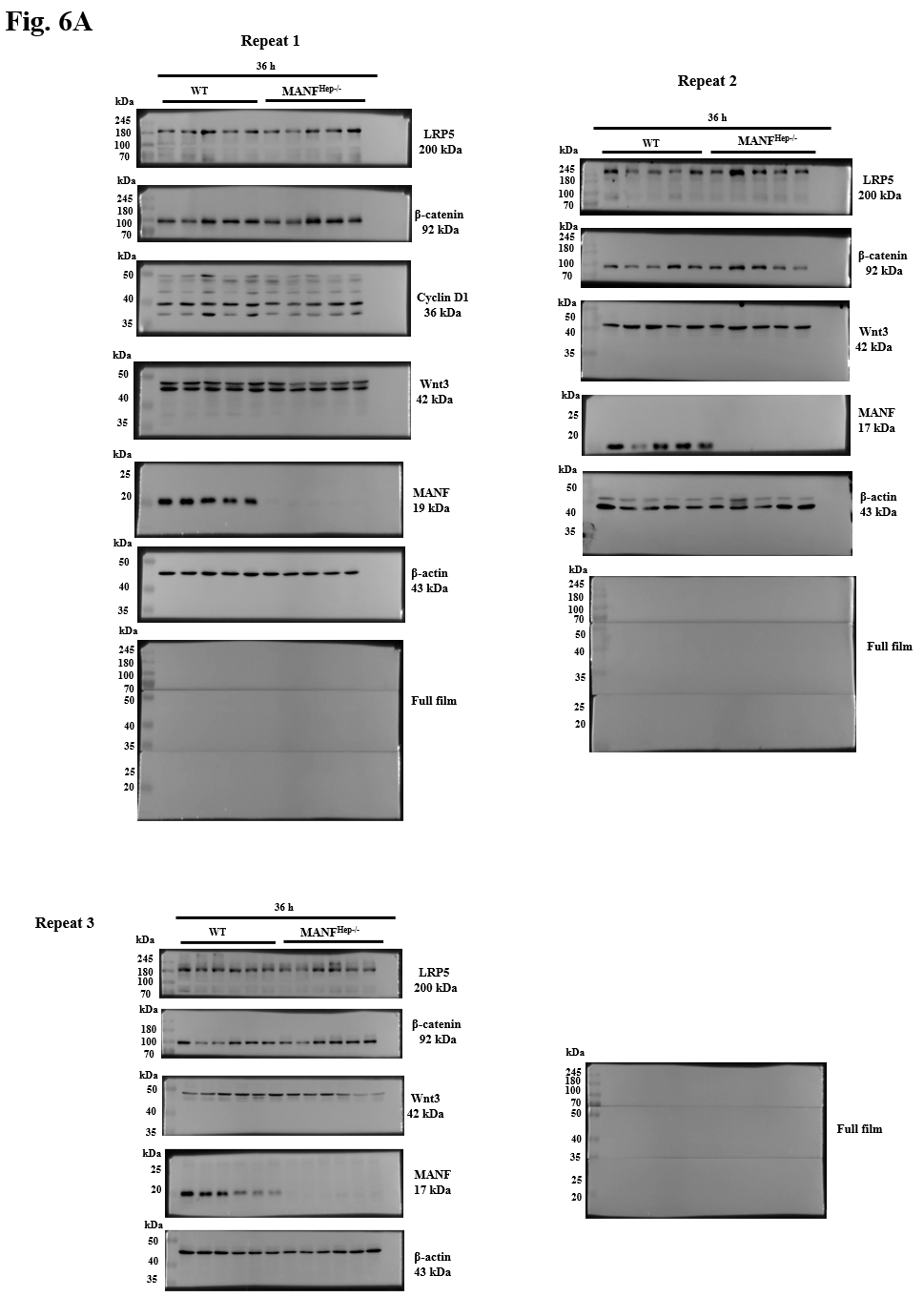


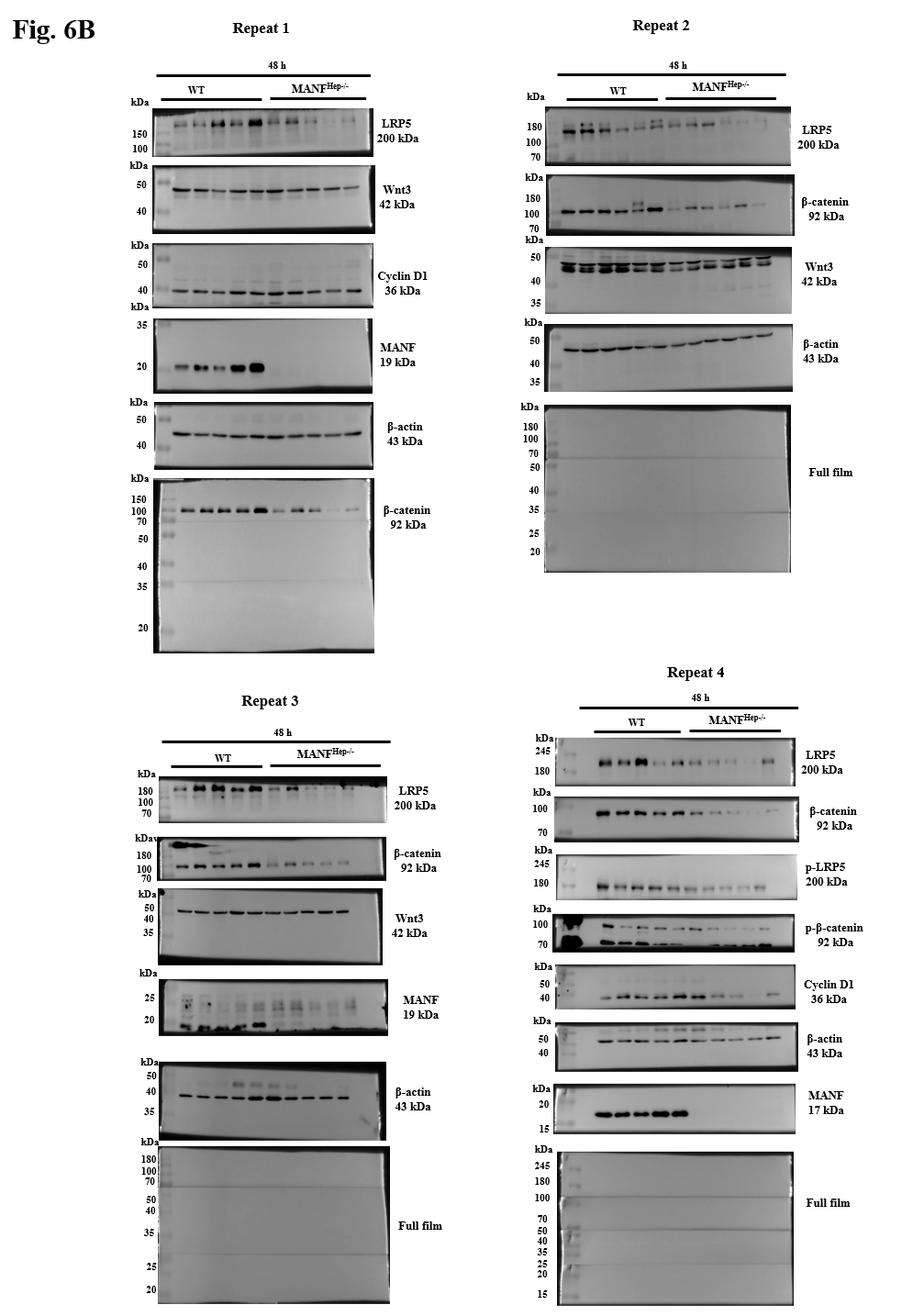


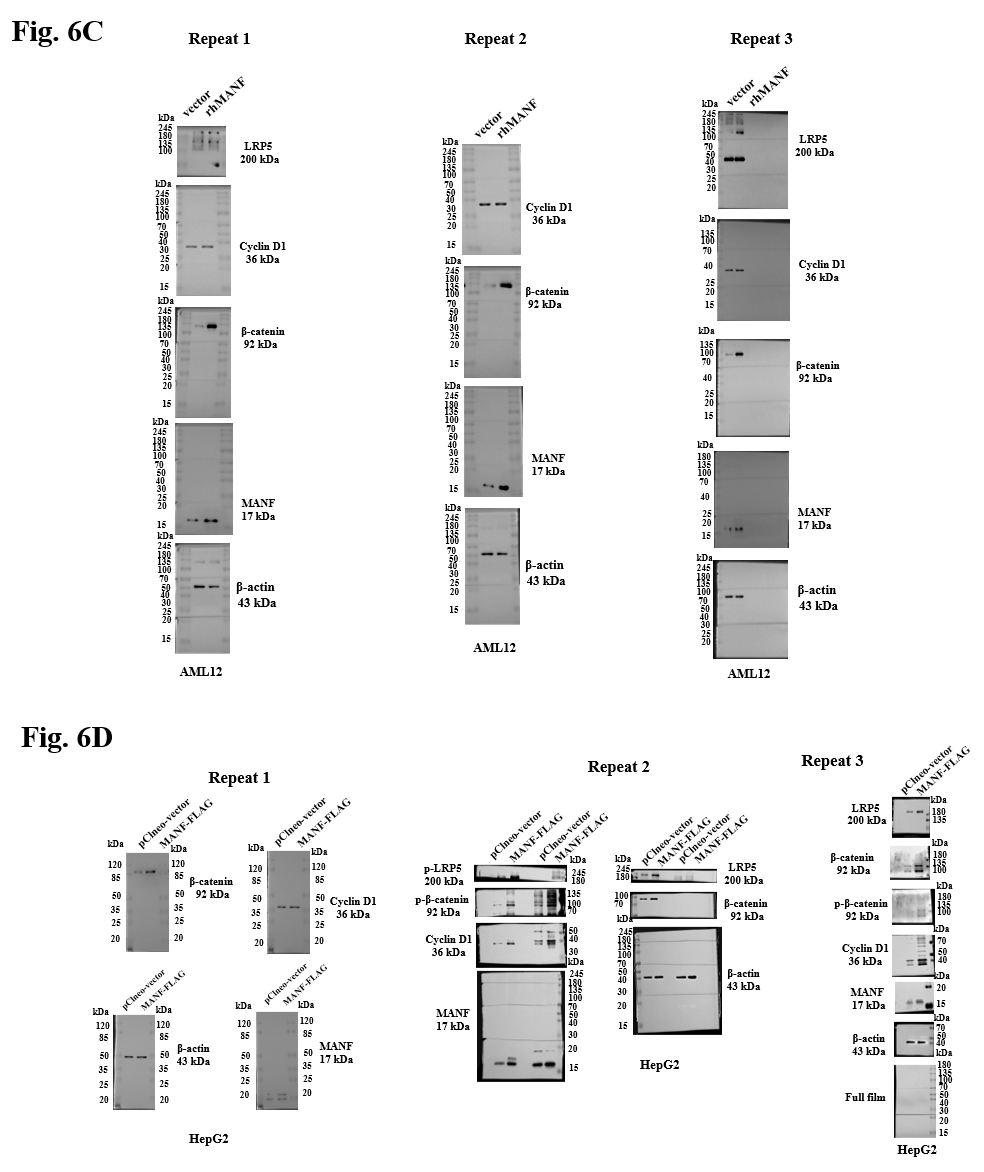


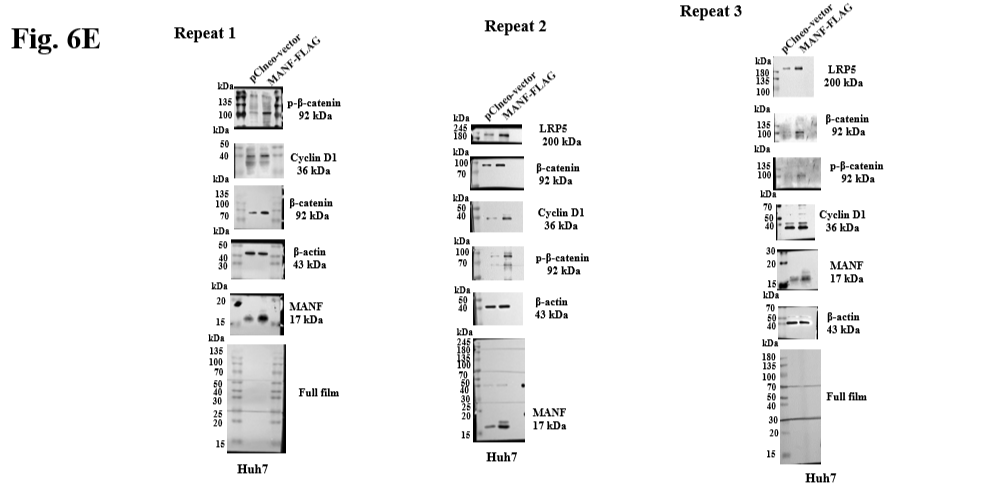


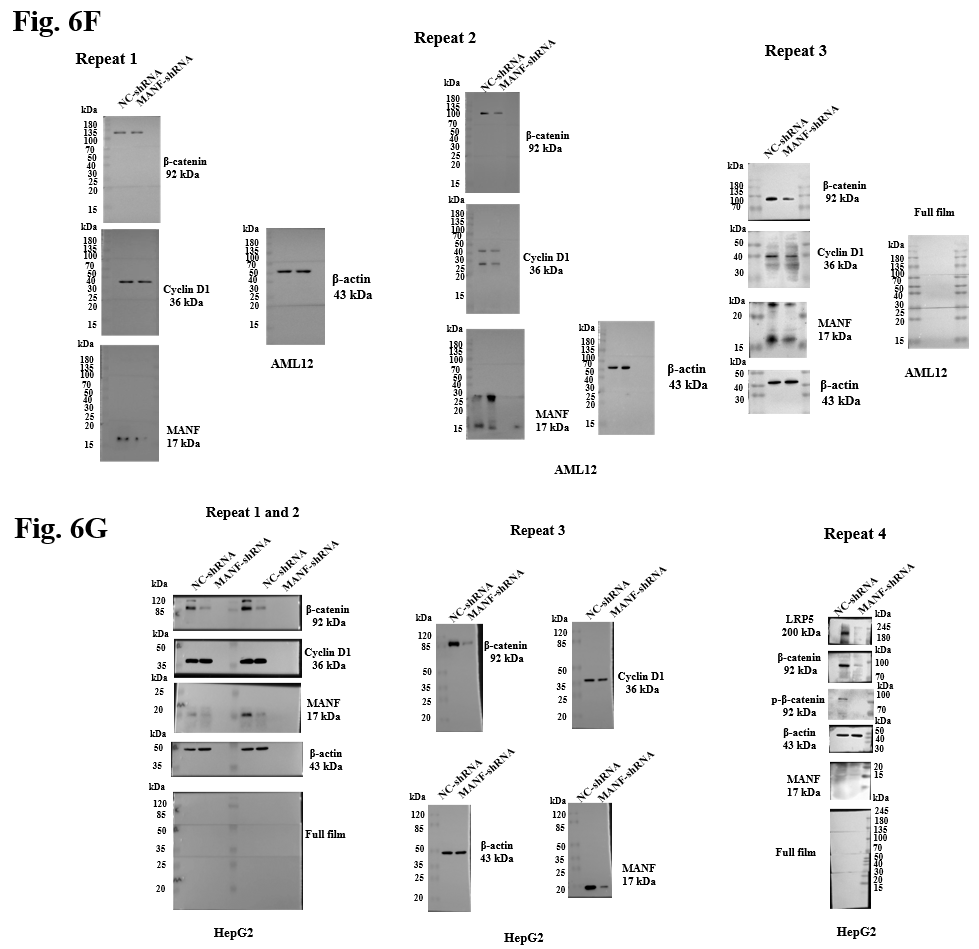


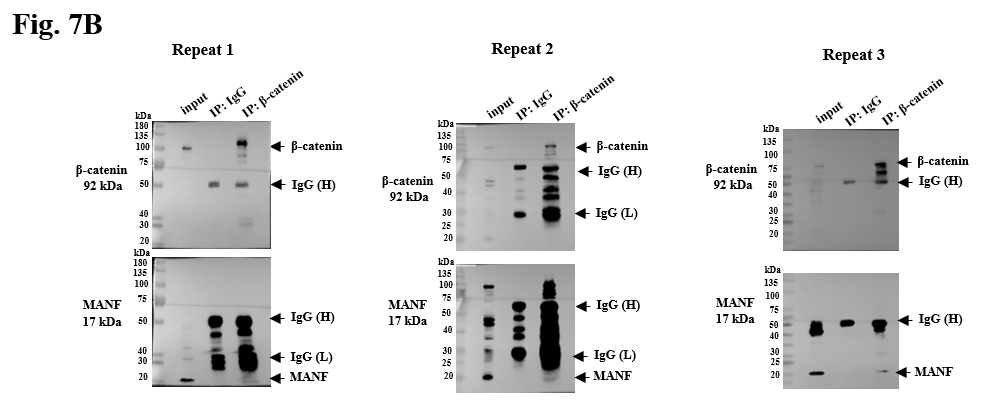


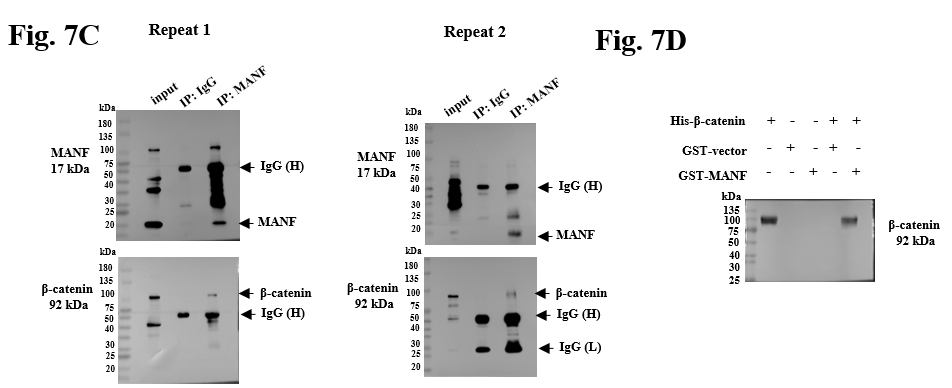


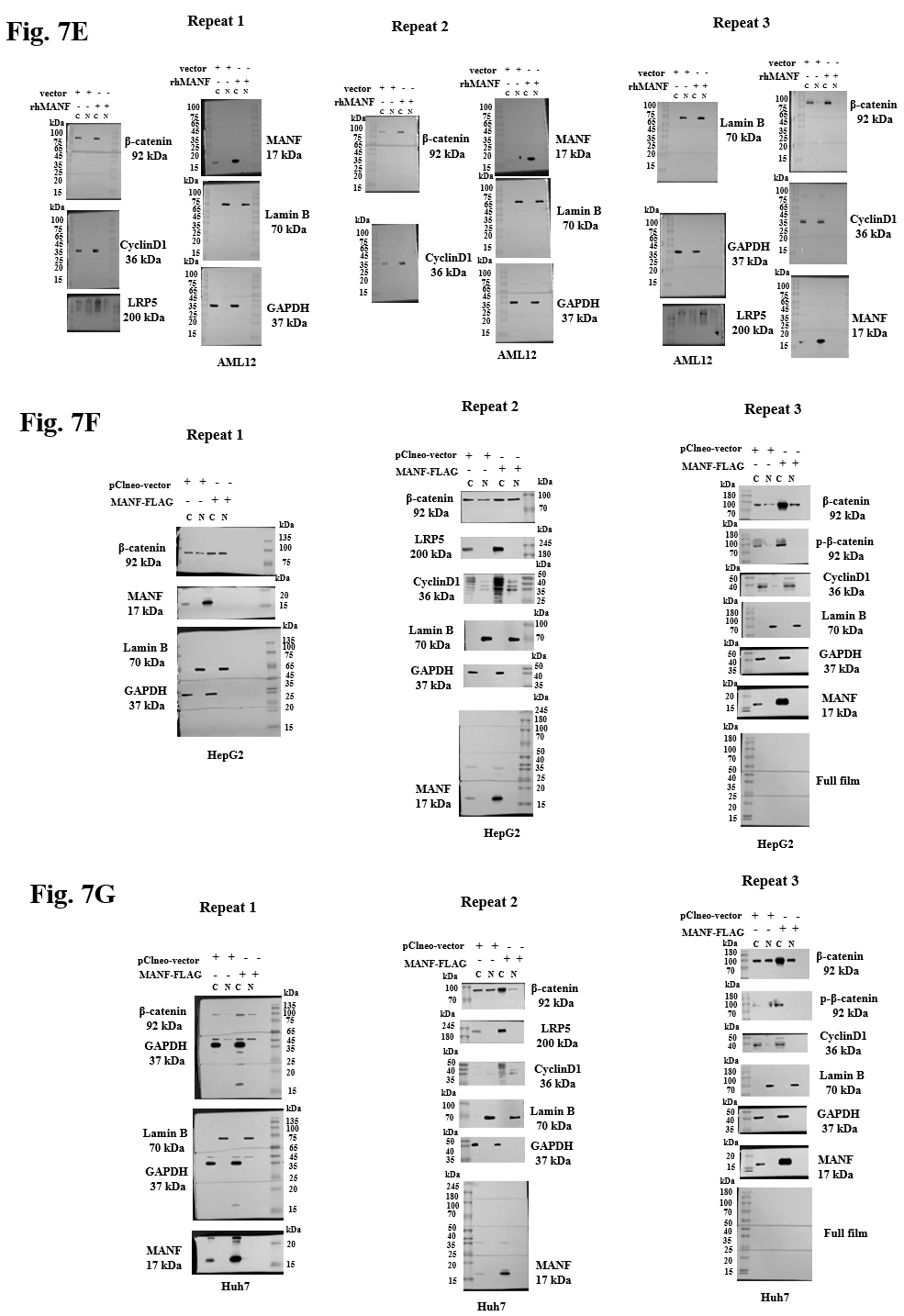


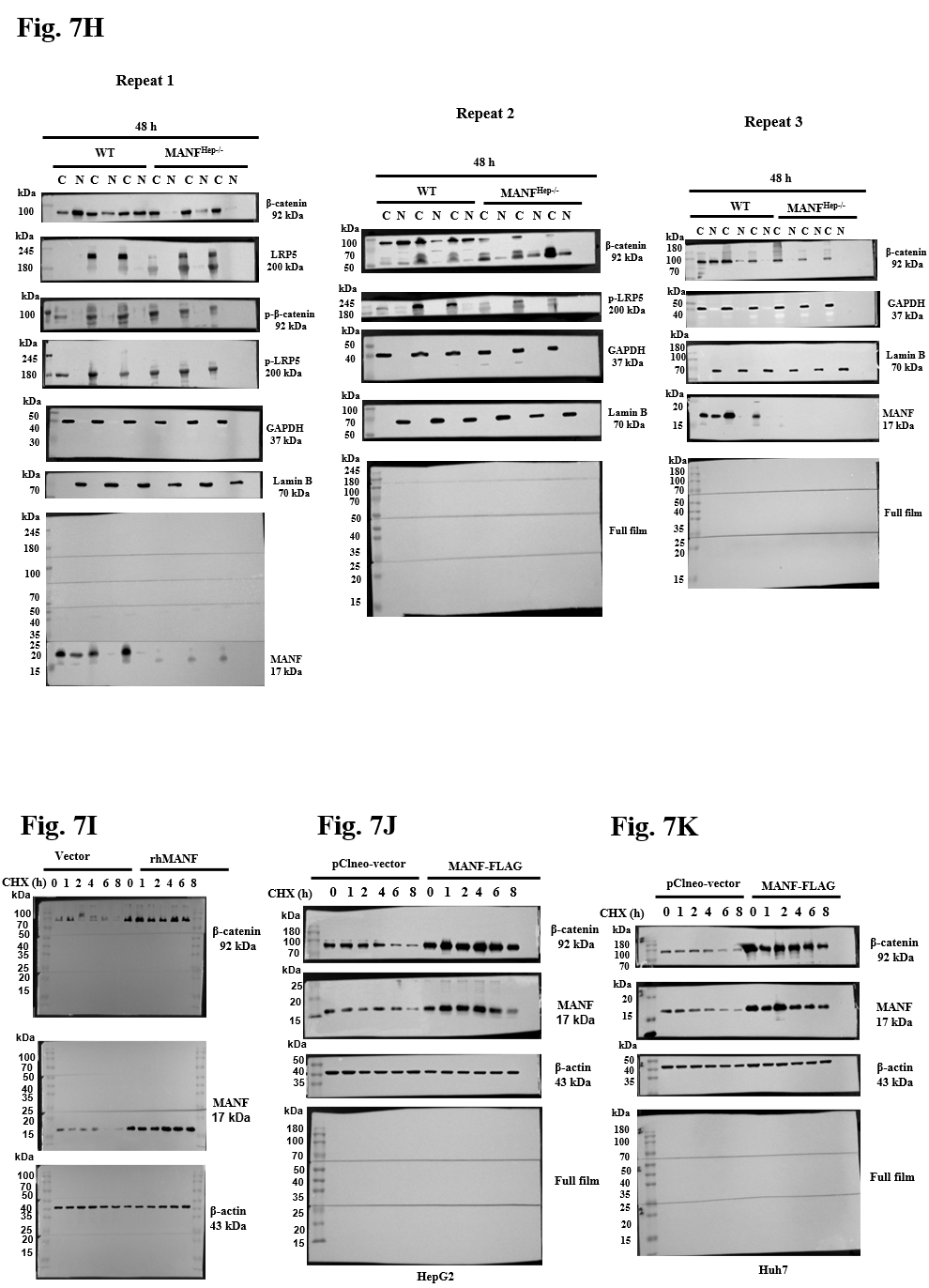


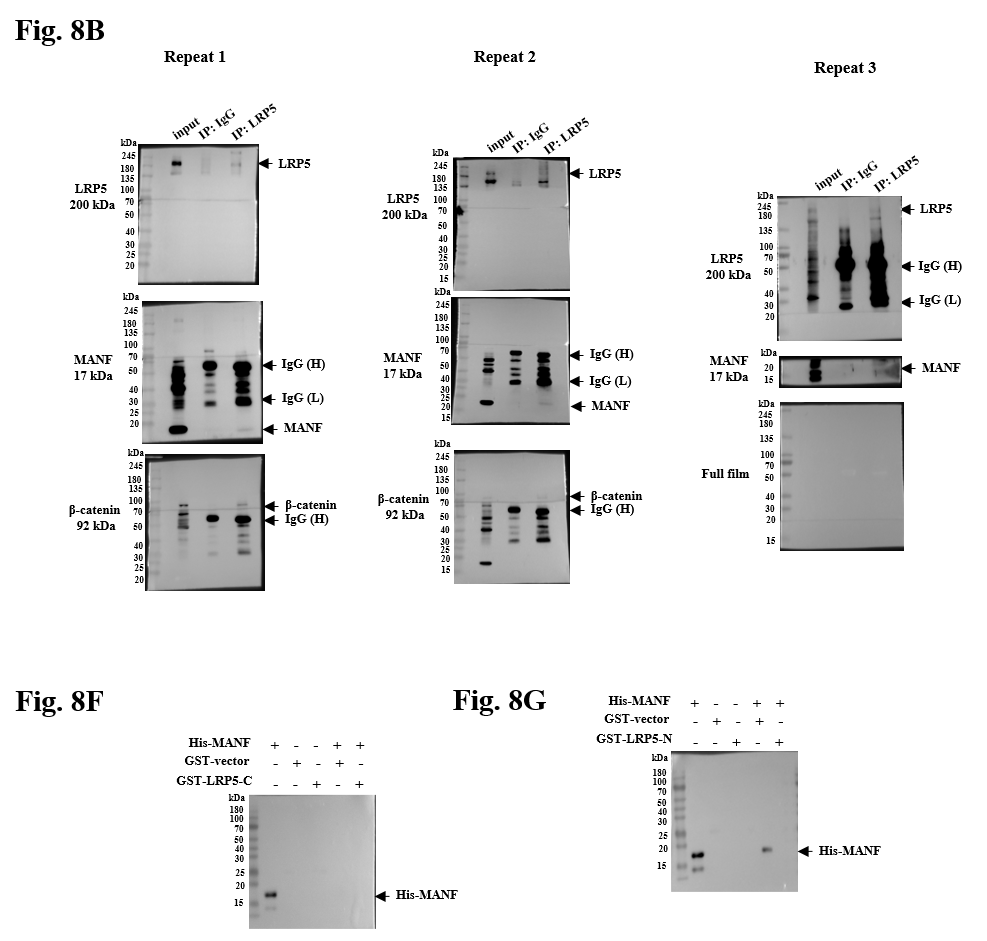


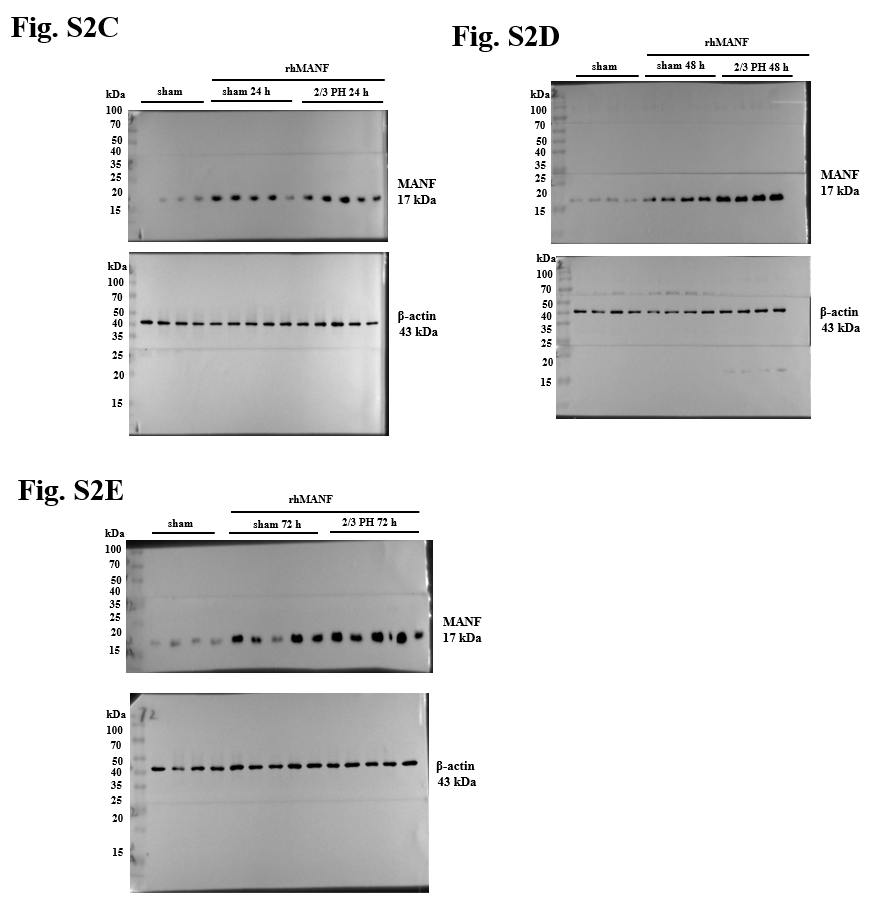


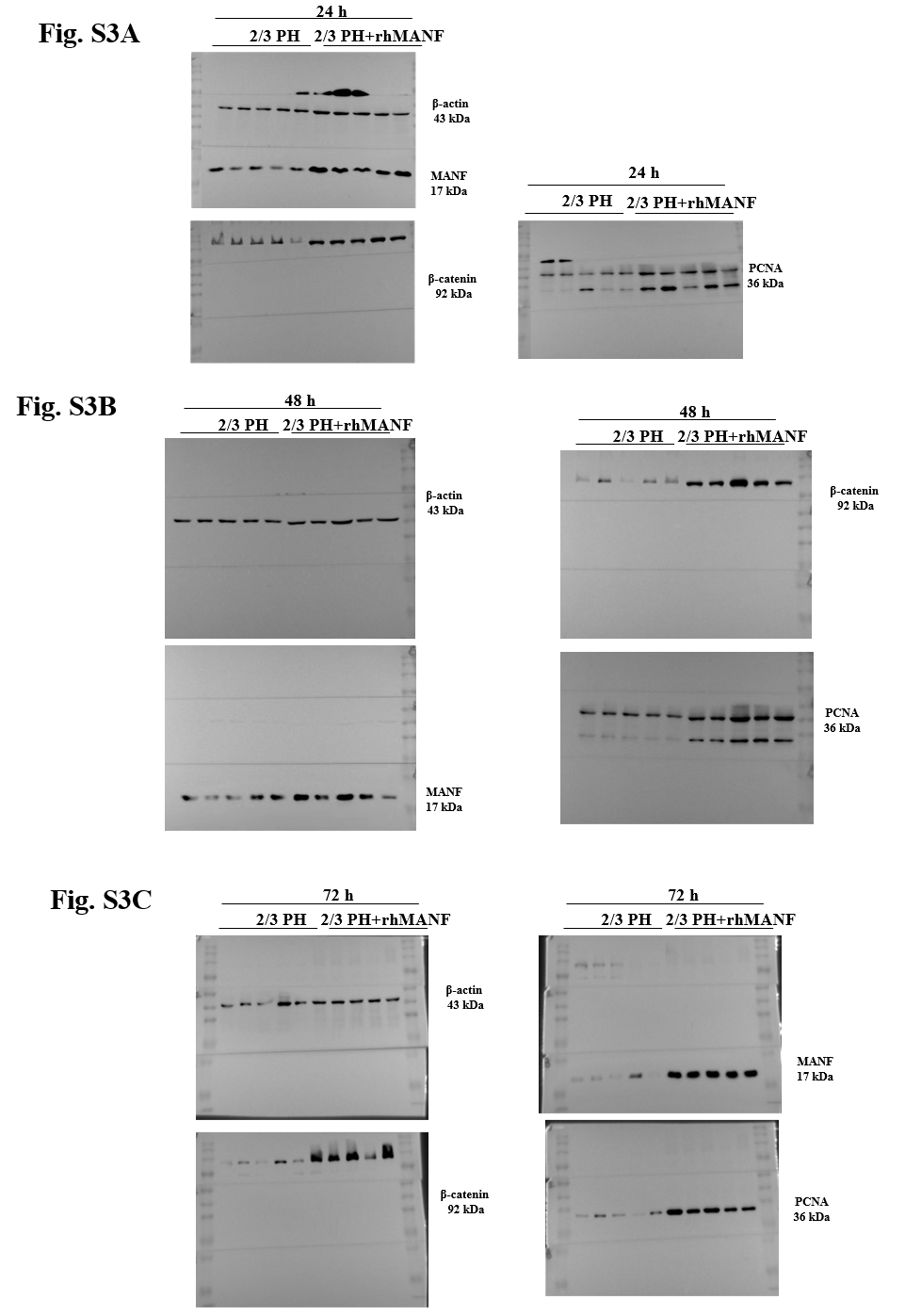


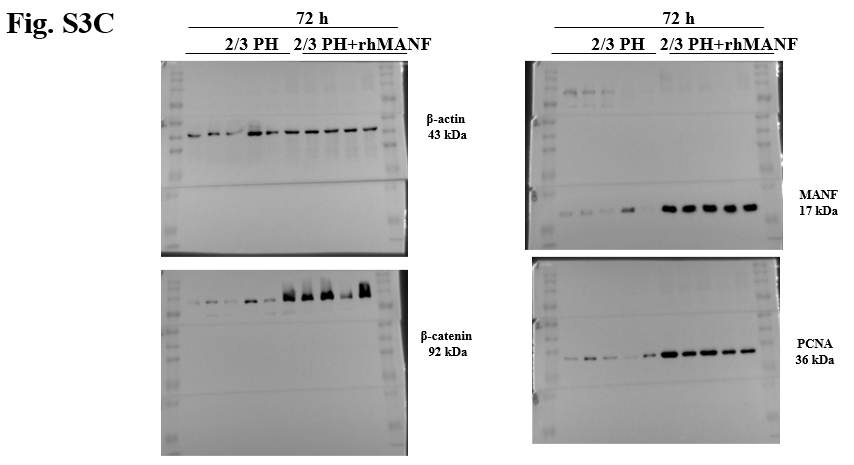


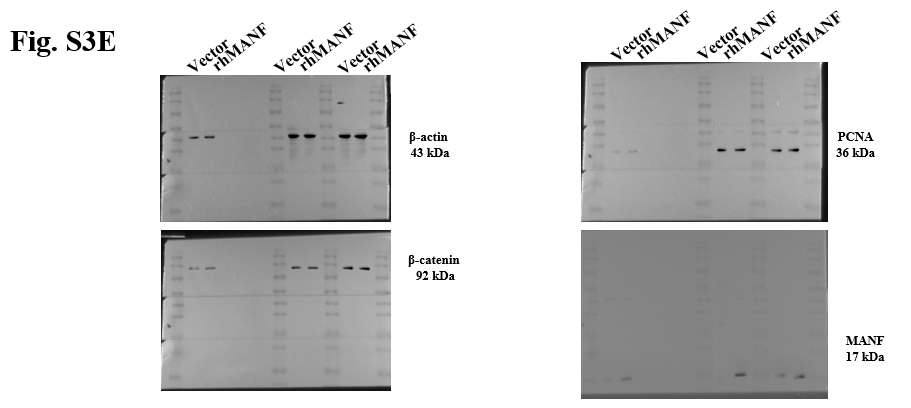


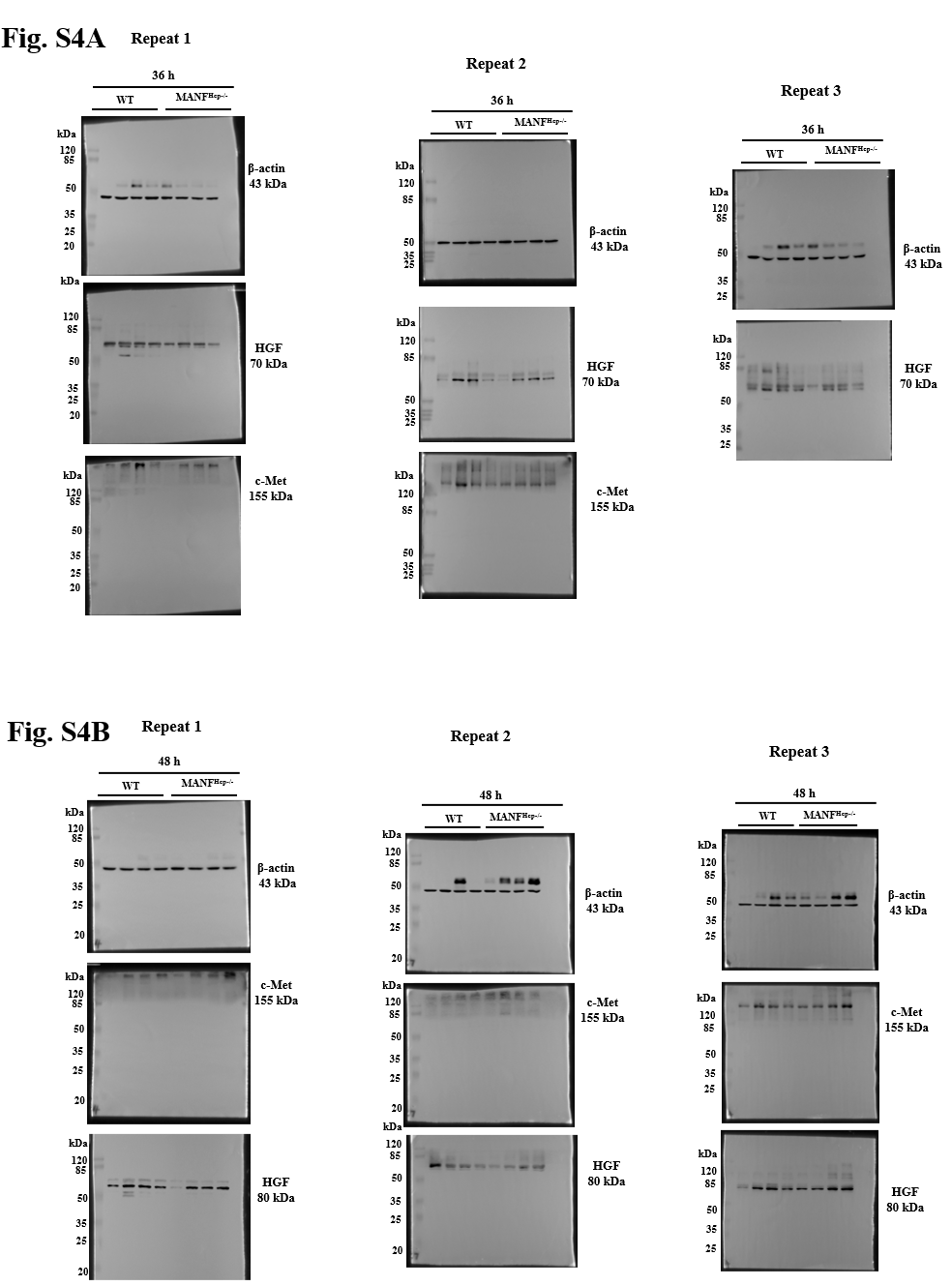

Supplement: Supplementary file 2 — original data from immunoblotting [file 41419_2024_7069_MOESM2_ESM.docx]
